# Tilly versus Milward: Experimental Evidence of Public Preferences for European Defense Amidst the Russian Threat

**DOI:** 10.1007/s11109-024-09979-x

**Published:** 2024-11-13

**Authors:** Alexandru D. Moise, Zbigniew Truchlewski, Ioana-Elena Oana

**Affiliations:** 1 Department of Political and Social Science, European University Institute, Badia Fiesolana - Via dei Roccettini 9, San Domenico di Fiesole (FI), Italy; 2 Department of Political Science, University of Amsterdam, Nieuwe Achtergracht 166, 1018 WV Amsterdam, Netherlands

**Keywords:** Public opinion, EU polity formation, Russian invasion of Ukraine, External security threat, J-Curve

## Abstract

Following the “bellicist” school of state formation, the external threat of war is expected to spur polity formation by centralizing military capacity (Tilly, in Coercion, Capital, and European States, Oxford, Basil Blackwell, 1990). It has been argued that Russia’s invasion of Ukraine could provide such an impetus for centralization in the EU polity (Kelemen & McNamara, Comparative Political Studies, 55(6):18–34, 2022). We adapt the Tillian argument to the era of mass democracy, where governments need citizen support. Public support is crucial because it can constrain governments in times of crisis, especially regarding salient policies. We do not yet understand what degree of centralization the European public supports and under which conditions it can increase. We conduct an experiment where we vary both the Russian (escalation from presence in Ukraine to the invasion of Moldova or Lithuania) and the American responses (continuation of support vs. withdrawal) and see how European preferences vary for polity building in defense. We field our experiment in 7 countries (Germany, France, Italy, Portugal, Finland, Poland, and Hungary) with different sensitivities and exposures to the war in Ukraine. We propose an alternative argument to the Tillian approach based on the seminal Milwardian argument according to which polity coordination of national capacities is preferred (Milward, in The European Rescue of the Nation State, University of California Press, Berkeley and Los Angeles, 1992). We show theoretically and empirically that external threats can actually hamper polity centralization, at least in the short term. Rather, they strengthen the subunits of a polity through coordination.

## Introduction: War, the Security-Efficiency Dilemma and Demand for Polity Formation

The formation of the European polity is a complex process in which crises play a key role. They can either trigger polity centralization (where a polity pools resources and policy making at the center to realize economies of scale and streamline decisions, through a new capacity like a common budget or a joint army), reinforce coordination of subunits within the polity (where subunits agree to certain rules of coordination, how to contribute existing resources and share the costs but retain their sovereignty), or simply cement the status quo with crisis responses being implemented at the level of subunits (for instance, each subunit decides to increase its pandemic or defense budget). When it comes to defense, we do not have a good understanding of what degree of centralization European citizens prefer and under which crisis circumstances they are more likely to support it. In the era of mass democracy, it is crucial to understand what shapes public preferences and, therefore, how they may enable or constrain polity formation. In this article, we connect the literature on political behavior, state formation, European studies, and international relations, and bring insights from a survey experiment on public preferences for defense centralization in order to shed light on these questions.

The argument of external crises inducing polity centralization rests on the state-building literature (Riker, 1964b). Most memorably, Charles Tilly wrote that “War made the state and the state made war” (Tilly 1975, 42). Recently, Kelemen and McNamara (2022) picked up on this “bellicist” approach to argue that the asymmetric European polity formation (strong regulatory powers, weak core powers) is due to its focus on the internal market and the lack of external threats. The implication is that the current Russian invasion of Ukraine and the threat of escalation is expected to spur EU polity formation and centralization of capacities by European policymakers. The need to fund war and compete more broadly against other polities in the international sphere is a powerful incentive to centralize tax collection and funds for purchases with economies of scale. This approach is focused on the supply side of politics (policymakers), and does not have much to say about public demand or support for polity centralization. However, we know that a strong dissensus among European publics can constrain further political integration (Hooghe & Marks, 2009). Conversely, a strong consensus allows greater room for maneuvering for politicians to strengthen the EU polity. Understanding public support for centralization in the defense realm is particularly important in a moment of such high salience^1^ for military policy. The present moment is thus an opportunity to probe the dynamics of public opinion on foreign policy at a time when the public is particularly attuned to the Russian invasion of Ukraine and the response of the EU.

We therefore ask what citizens are willing to accept in terms of centralizing military policy in order to see what constraints policy-makers face. This paper diverges from the “Tillian” and bellicist approach to EU polity formation. First, it contributes by focusing on the demand side of politics (public opinion) to see whether the same mechanism applies and whether announcements made by policymakers are mirrored by European public opinion. The Tillian thesis was developed to explain state formation in the Middle Ages and Renaissance when elites were insulated from the public. Modern democratic nation-states need to take public opinion into account, as it may constrain or enable elite action. Furthermore, public opinion is especially important in defense policy. Studies of preferences for fiscal spending indicate that respondents are especially polarized on defense spending between left and right and that defense spending is usually seen as a first candidate for cuts during austerity (Huebscher et al., 2023). There is thus a dissensus that limits the margins for maneuvering at the EU level.

Second, we argue that the EU faces a steep “J-Curve” of polity building, i.e., with support decreasing in the short-term but increasing in the long-term, in case of an external threat, due to its very nature. The EU is a compound polity where member states are “policymakers of last resort”, especially in the defense realm, because this is where most of the capacity lies. Consequently, for Europeans, coordination may be seen as less costly (politically and economically) in the short run than European capacity building and political centralization (which can give rise to feelings of lost sovereignty among EU citizens). Additionally, the EU is heavily reliant on US security guarantees as most of its member states are also NATO members (in our study we also include a non-NATO member, Finland). Consequently, we argue, the external threat of war may, in the short run, push respondents from member states to prefer coordination over centralization, in line with a Milwardian approach to EU polity formation (Milward, 1992). We develop a theory that has both short-term and long-term implications. In this article, we test the short-term, the beginning of the J-curve, leaving the long-term implications to be tested by future research.

Accordingly, the linkage between war and polity building is more complex in the EU and this makes it a special case to study Tilly’s approach. While in Tilly’s approach the supply side (the elites) may drive polity formation and centralization in case of war, we need to know whether the demand side (individual-level preferences) would enable such policymaking steps at the EU level. For these reasons, the contribution by Kelemen and McNamara (2022) has triggered a debate on whether or not external threats should spur EU polity formation (Genschel & Schimmelfennig, 2022; Freudlsperger & Schimmelfennig, 2022; Eilstrup-Sangiovanni, 2022). As others have noted, in the case of the EU the war is on its doorstep not on its territory, while its security is already guaranteed by NATO and already-developed national infrastructure (Genschel, 2022). Contributions from the direction of the “core state powers” literature especially stress that European state building is unlikely because national elites would have to abdicate core state powers at great material and public opinion costs (Genschel & Jachtenfuchs, 2014): overcoming the regulatory polity only happens in the face of massive functional pressures. Nonetheless, the EU involvement in the war is profound (McNamara & Kelemen, 2022), while evidence shows that EU citizens do not think that EU security should come at the expense of NATO security, but rather want both (Wang & Moise, 2023).

Our aim is to contribute to this debate by clarifying the causal route through which external threats and war in this case impact EU polity formation, and to bring demand-side experimental evidence on individual-level preferences to the table, shedding light on the constraints governments face. We thus utilize an experiment to understand whether European individuals privilege security or efficiency. To be sure, while the “Tillian” theory is state-level, we open the causal box and look at the demand side. We fielded our experiment in July 2022, a moment of very high salience for the Russian war in Ukraine and the economic and political response of the EU. Thus, while foreign policy is usually considered too complex for individuals and of low salience, our timing allows us to test public opinion at a time when the public is aware and engaged in foreign policy discussions. Indeed, several elections, such as those in Hungary^2^ and Slovakia^3^, showed that policy on the war was crucial for electoral success and that elites need to listen to public opinion.

The paper proceeds as follows. The next section elaborates our theoretical expectations regarding individual and country-level preferences for polity centralization or coordination in case of an external threat, leveraging the concept of the J-Curve. The third section presents our data and the context and the fourth section presents the results. We conclude with a discussion. Overall, we show that, when faced with an external threat like war, following our argument based on the J-Curve, Europeans prefer strong coordination to the centralization of the EU polity through an EU army. This has important and paradoxical implications for Monnet’s dictum on how the EU will be shaped by crises.

## The EU Polity, External Threats and the Curse of the J-Curve on the Demand Side

In case of an external threat, when highly salient policies take center stage in the public sphere, consensus on the demand side becomes crucial for passing policies. What do citizens want and how can this democratic constraint on policymaking enable or disable polity formation? To answer this question, we leverage the concept of the J-Curve, used widely in political economy to illustrate the calculus of crisis and reform broadly understood (Gans-Morse & Nichter, 2008; Hellman, 1998; Magee, 1973). Simply put, the J-Curve stipulates that the costs of reforms are greatest in the short run and very much concentrated in the initial stages of reform, while benefits only materialize in the long run. This creates a J-Curve of cost and benefits that sheds light on why the political economy of reforms is so difficult. We develop the concept of the J-Curve as it applies to the EU’s security dilemma following the Russian invasion of Ukraine, focusing on how characteristics of the J-Curve should affect public preferences for security policy.

Crucially, the shape of the J-Curve depends on two parameters, which we name the “intercept” and the “slope” conditions (Genschel, 2022; Kelemen & McNamara, 2022). The intercept condition refers to the initial conditions of a polity and how they affect the calculus of reform, for this paper military centralization, military cooperation, or unilateral actions. In our case, the intercept of the J-Curve (the structure of a polity) is shaped by two factors: whether its subunits are strong or weak, and whether it has external security guarantees. These initial conditions shape the associated costs and benefits of centralization and, thus, initial preferences for centralization and new capacity building versus coordination or unilateral action. The comparative advantage of centralization is more likely to materialize in polities with weak subunits and no external security guarantees while cooperation or unilateral action is more likely in polities with strong subunits and security guarantees. For the former, the J-Curve shifts up, while for the latter it shifts down.

The slope condition refers to changing preferences of the demand side for polity centralization versus coordination or unilateral action (Genschel, 2022; Hooghe et al., 2019). It depends on two factors: the size or intensity of the threat, and the removal of external security guarantees. For this reason, we conceptualize it as the slope of the J-Curve: the higher the threat to the polity (either due to escalation of the conflict or to loss of security guarantees), the steeper the J-Curve and the faster the preference will change from unilateral action to coordination and centralization. We adapt the J-Curve to the problem of external threats and polity formation and show how different initial conditions change the calculus of polity formation between centralization and coordination when an external threat arises.

### The J-Curve: Tillian vs. Milwardian Polity Formation on the Demand Side

We contrast two arguments on demand side side preferences for polity formation in times of threat. On one side, the “Tillian” view implies that external threats should lead to preferences for polity centralization and transfer of capacity from the subunits to the center (Kelemen & McNamara, 2022; Cederman et al., 2023; Kenkel & Paine, 20232; Tilly, 1990, 1985; Riker, 1964a). On the other, the “Milwardian” view indicates that in compound polities, subunits of a polity are states with considerable capacity in core state powers and that in case of threat the center merely enhances national capacities instead of transferring them to the center (Milward, 1992; Genschel & Jachtenfuchs, 2014; Schelkle, 2017).

First, the “Tillians” would argue that in a given polity with subnational units external threats and wars create pressures for centralization through the “security imperative”. This applies even if the different national subunits prefer coordination of existing capacities rather than centralization and the creation of new polity capacities before the external threat arises.

To see why there is an alignment of interests between the national subunits and the central government to centralize further and create new capacities at the polity level, it is important to take into account that in the Tillian approach national subunits are weaker than the collective polity. Consequently, the common good of collective survival can be most efficiently guaranteed by pooling common resources and streamlining decision-making processes around one goal (i.e., solidarity and centralization).

**Fig. 1:**
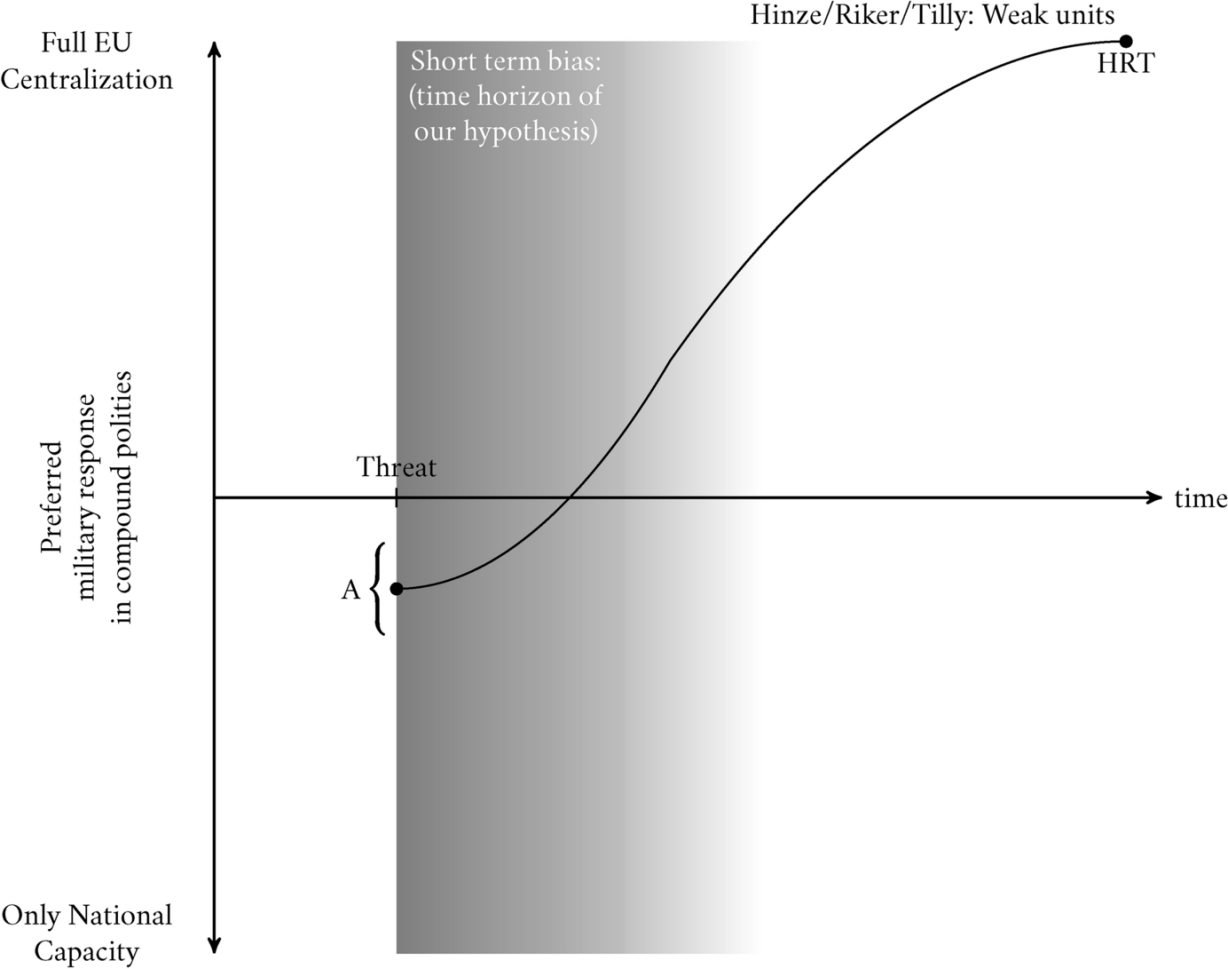
The J-Curve in Tillian/Rikerian Polity Formation

Figure 1, and subsequent figures, illustrate our theoretical argument. On the *y* axis we plot preferences for centralization on a continuum ranging from full EU centralization of military capacity (top) to maintaining only national capacity (bottom). In between there is a broad spectrum of types of coordination. On the *x* axis we plot time. The start of the gray area denotes the start of the threat. The starting point of our analysis is the initial preference for polity centralization, denoted by point *A* in the figure, our intercept. Here we illustrate the “Tilly-Riker” scenario, of a polity with weak subunits. In normal times, subunits prefer to maintain their autonomy (hence the low starting point of the intercept A). However, once the threat manifests itself, preferences (elite in their case, public in ours) quickly shift towards more coordination and centralization. The gray area denotes the time horizon of our study. Thus, even in a short time horizon, weak subunits want to centralize in order to manage the threat. The benefits of centralizing capacity accrue quickly while the costs of inaction are high.

The reasoning is straightforward. The benefits of centralization among weak subunits outpace the benefits of unilateral action or mere coordination, due to economies of scale and lower transaction costs. This is the assumption that in early state-formation weak subunits can find it easier to centralize than to cooperate, while facing existential risks if they do nothing. Hence the argument that wars make states through tax creation, bureaucracy development, and army creation. This is because common threats to a polity as a whole result in an exceptional period where elites and citizens acquiesce to polity centralization, especially when technological advances require heavier investments into collective defense. Increasing returns to fiscal and geographic scale also come from political economic exchanges between the protectors at the borders and the protected in the hinterland (returns to scale may also have limits, see, for example, Boix et al. (2011)). These exchanges propel the centralization of the polity through taxation (Mangini & Petroff, 2022; Scheve & Stasavage, 2010). Importantly, external threats create pressure for centralization even if the threat stays constant. This is because the asymmetry between weak subunits and a centralized foe compound over time.

The implication of Kelemen and McNamara (2022) is that an external threat to the European Union (EU) would spur centralization of military capacity just as the “bellicist” argument would have it. *Prima facie*, this seems to make sense: various prominent voices have argued that a defense union is the only possible answer to the Russian invasion of Ukraine, to the resurgent military threat from Russia and to the strategic competition from China (Braw 2022; Herr & Speer 2022, for a sample). The argument is Tillian: EU member states will require greater coordination and centralization to face threats from highly centralized geopolitical competitors. The EU is already facing unity problems as dissenting governments, such as Hungary, have stalled sanctions on Russia and aid to Ukraine. The Juncker Commission started the European Defense Fund in 2019 when the European executive identified external threats to its sovereignty (Haroche, 2020). Although still small compared to the EU’s economic capacity, the fund is developing fast and builds on other, earlier initiatives like the creation of a European Defense Agency in 2004, after the wars in Irak and Afghanistan (Bátora, 2009).

We can expect these same mechanisms to impact the demand side of public opinion. We note that public opinion is more likely to exert pressure on politicians during times of high salience, such as the pandemic or the Russian invasion of Ukraine when voters follow what is going on and express preferences. In a direct manner, when faced with threats, individuals demand security. When they understand that the lower-level political unit cannot provide enough security, they shift their preferences for greater coordination or centralization. In an indirect mechanism, individuals follow political elites and inform their preferences based on the cues they receive. Thus, public opinion can act either as a push factor for centralization or coordination when there is consensus and high demand, or as an enabling (or constraining) factor in other scenarios.

There is evidence to believe that, similarly to the pandemic (Altiparmakis et al., 2021), the Russian invasion of Ukraine triggered a rally-around-the-flag moment among European voters, who became more supportive of leaders and policies following the start of the war (Truchlewski et al., 2023; Steiner et al., 2023). Importantly, this implies that EU elites have greater room for maneuvering in order to pursue greater integration. Conversely, if far-right parties are not able to capitalize on possible dissensus, they may prove unable to create political momentum against such policies, as postfunctionalism would predict (Hooghe & Marks, 2009).

**Fig. 2:**
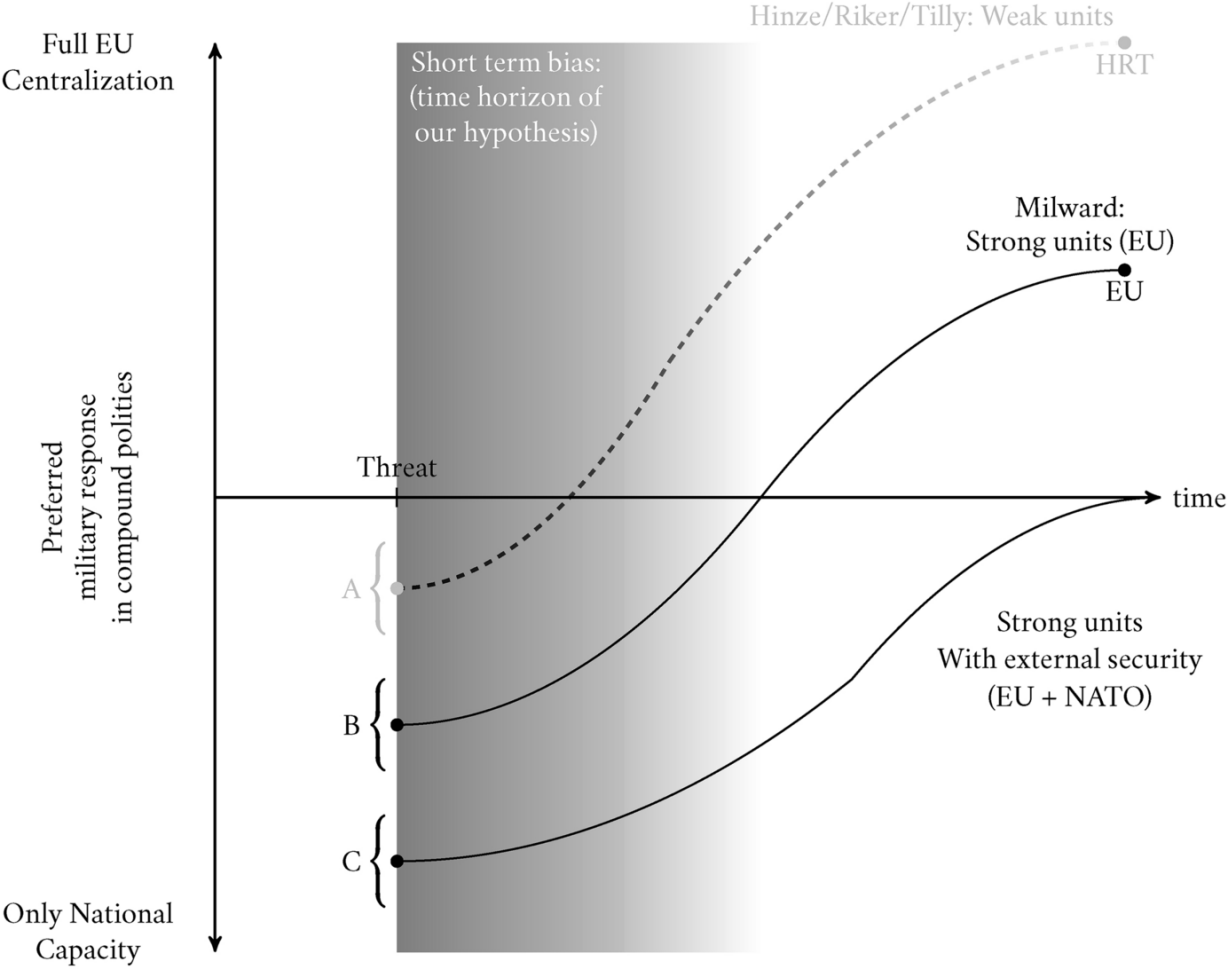
The J-Curve and the Intercept Condition (Polity and Security Structure)

### The Intercept Condition: the Milwardian EU Polity and its Security Structure

There are, however, several key differences between the EU and the polities that formed in the Middle Ages and the Renaissance, which change the intercept of the J-Curve in Figure 2 and shift it towards national capacity or cooperation rather than centralization, at least in the short run (From *A* to *B* and to *C*). This is what we call the “Milwardian” polity formation.

The first important fact is that the EU is a compound polity formed of mature national states. This very fact changes the calculus of polity formation in case of a threat because polity subunits are not weak anymore, but strong. Each EU member state has its own fully developed budgetary apparatus that underpins a professional, long-standing national army. The implication is that the cost of centralization and new capacity building at the center increases, while the benefits decrease. In the face of a threat, even strong states can benefit from pooling resources but they are more likely to do so through coordination, utilizing existing national infrastructures rather than building new capacity. This is because transferring subunit capacity to the polity-wide level entails political and economic frictions, especially in the short run. Heterogenous capacities and path dependencies at the subunit level entail costly negotiations about centralization that cannot easily be sorted out when the threat occurs. We therefore move from point *A* to *B*: an overall lower level of preferences in the direction of centralization irrespective of the nature of the threat, that is, a lower intercept irrespective of the slope.

The second important fact that changes the intercept of the J-Curve is the security organization in which the EU is embedded. In contrast to the first scenario starting at *A*, most EU member states are also members of the North Atlantic Treaty Organization (NATO) which, on top of increasing the benefits of coordination through institutionalization (moving the J-Curve to point *C*), also entails the security guarantees of a dominant player, the United States. Genschel et al. (2023) argue that US security guarantees fulfill the need of EU member states for supranational insurance, and thus prevent EU capacity building in defense, despite a permissive consensus of public opinion. We argue that public opinion itself is shaped by such guarantees. Directly, EU citizens should perceive less threat from Russia, given NATO security. Indirectly, political elites are less likely to cue their supporters that additional security structures are necessary. Thus, external security guarantees push the intercept lower still, to *C*.

Our intercept condition offers important insights into polity-building. In general, once exposed to an external threat like war, Europeans will prefer to see cooperation over polity centralization through the creation of a common EU army. This stems from the substantial political, economic and administrative costs of such a step: respondents may consider the creation of an EU army a daunting if not unrealistic task, precisely at the moment when defense needs to be most effective, hence the fall-back onto coordination rather than centralization. Even in the long term, without a powerful enough threat, there is little incentive to build up European level security structures if NATO security can be relied upon with absolute certainty. In reality, NATO security guarantees are not absolute. Unlike a centralized polity with a single army, sovereign states belonging to a treaty can make political decisions that go against the interests of the alliance, as shown by Trump’s comments undermining NATO^4^ and by conflicts between Turkey and the US in the Middle East (Thompson, 2022). We therefore expect respondents to still be in favor of greater coordination and be least favorable to maintaining the status quo. A new threat requires new responses. Coordination need not be perceived as costly as centralization, nor that it should come at the expense of NATO guarantees.

We therefore formulate Hypothesis 1:

- **H1:** in a polity like the EU with strong subunits and external security guarantees, public preference for coordination will be higher than preference for centralization, due to its perceived costs.

**Fig. 3:**
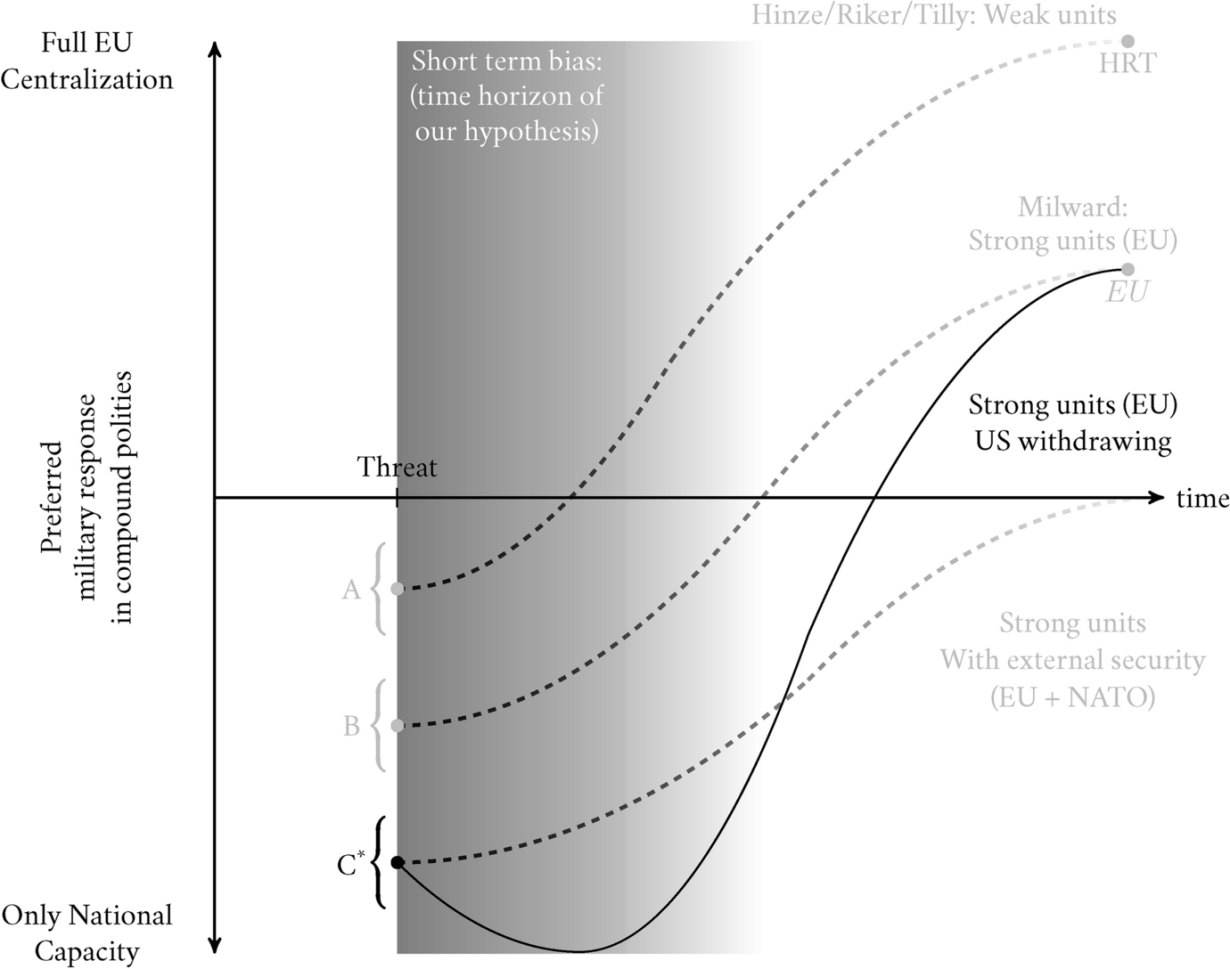
The J-Curve: the Intercept Condition with US Security Removed

### The Slope Condition: Changing Security Structure and Russian Escalation

We have so far discussed the factors affecting the intercept, that is the initial condition at the start of a threat. We now turn to the slope of preferences for centralization. The slope describes how, starting from the intercept, preferences shift as the threat continues. We describe two factors that can affect it.

The first builds on the intercept condition of external security guarantees. Past and current events like the election of Trump, the discourse on free riding in NATO, and the reluctance of Republicans to further fund Ukraine’s defense, beg the issue of what would happen if Europe’s security architecture changed with the withdrawal of support from the United States. Such a situation, which we illustrate in Figure 3, would not be akin to a simple shift from point *C* to *B* (i.e., a change of intercept), of strong national units without external security. The reason is that Europe is dependent on US security. Its withdrawal would create a short-term security vacuum with profound implications. The costs of centralization would increase in the short term. Centralization requires time to build infrastructure, both legal and material. A common army would need time to form and become effective. Such time is not available when the threat is active. An external security umbrella guarantees stability until the costs of centralization are outweighed by the benefits. In the absence of such external security the costs will outweigh the benefits in the short term as the polity needs to be immediately prepared. Thus, preferences would start from the same intercept, point *C*, but would shift down in the short term. In the long term, the need for security guarantees would outweigh the costs and the slope would, on average, reach the same point as the scenario starting from *B*, as if the external guarantee hadn’t existed. We thus formulate Hypothesis 2:

- **H2:** If the US withdraws from the Ukraine conflict, the immediate security vacuum implies a deepening of short-term costs and, therefore, a higher preference for using existing structures (national only or coordination) rather than building new structures (centralization).

**Fig. 4:**
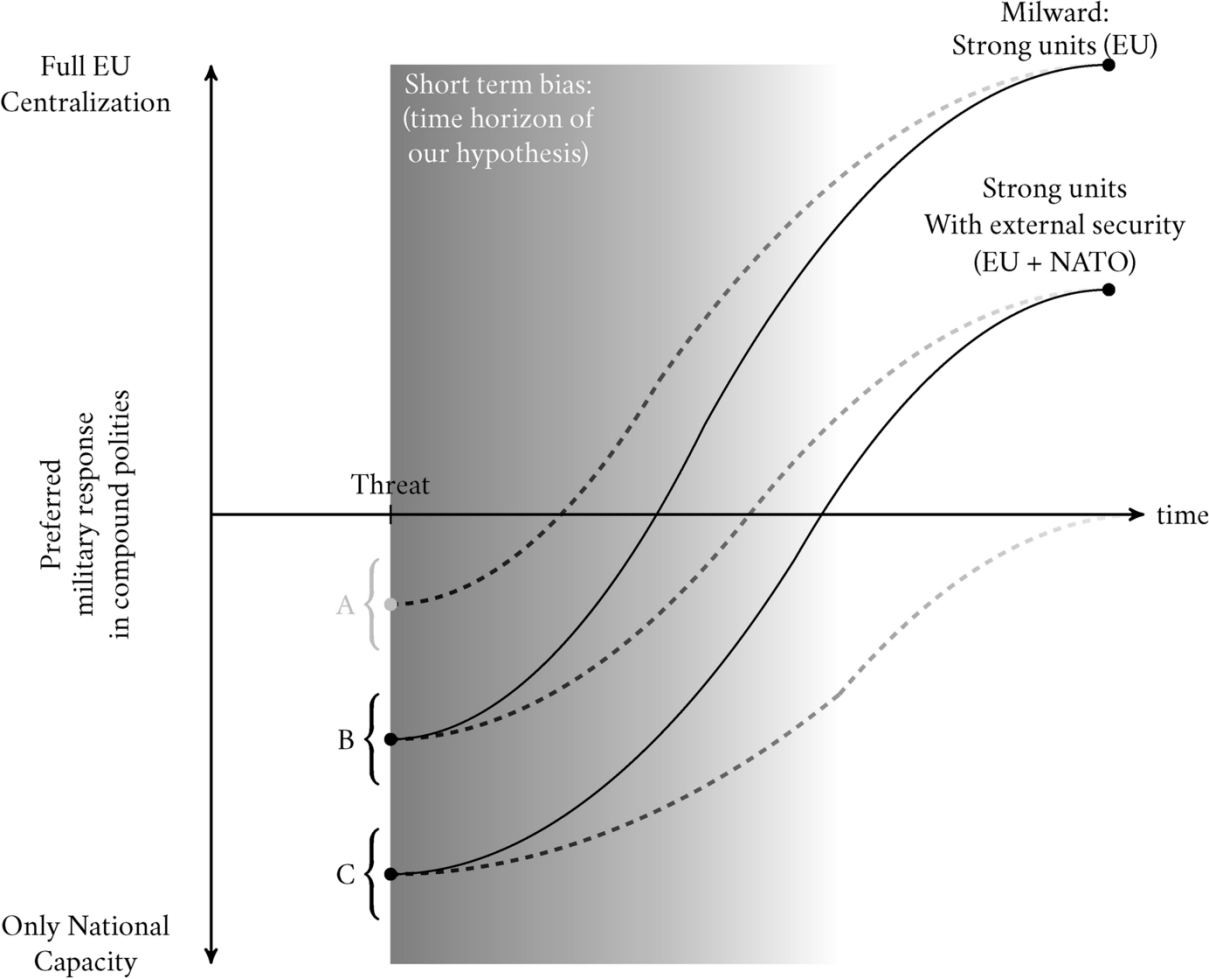
The J-Curve and the Slope Condition (Russian Escalation)

The other factor affecting the slope of centralization preferences regards the scale of the Russian threat. Similarly to the withdrawal of US security, the escalation of the conflict by Russia would change the slope of J-Curve: in Figure 4 increasing escalation of the threat shifts the slope up (solid lines) compared to the previous ones (dashed lines). Ceteris paribus, a higher threat should lead to stronger preferences for centralization. Even in the case of external security guarantees, a high enough threat should persuade individuals of the benefits of pooling more resources together. In the long run, a sustained high level of threat should convince individuals that even small amounts of instability in the security architecture are unacceptable and that security needs to be guaranteed by their own resources. Thus, our theory predicts that escalating the conflict would increase preferences for centralization in the long term. We argue that accountability problems and the shorter-term horizon of coordination will outweigh the delegation problems involved in centralization in the long term as the need for stable solutions becomes more pressing and the returns to scale more apparent. Furthermore, while the short-term patterns are subject to heterogeneity both between socio-political groups and between Member States, we expect a high level of sustained threat to the entire polity to decrease such heterogeneity in the long term as the need for stronger security guarantees outweighs sovereignty concerns. We leave these predictions to be tested by future studies, which can weigh the impact of such a scenario in the long term. We focus instead on the time horizon available to us at the time of our experiment (July 2022), the short term (the gray area in the figures).

We therefore formulate Hypothesis 3:

- **H3:** for polities with strong subunits, the escalation of the threat (Factor 1) will not result in increased preferences for centralization, in the short run, but will lead to increased preferences for national and coordinated solutions.

### Heterogeneity of the J-Curve

Our J-Curve argument has so far focused on the macro conditions (threat level and external security) that are likely to influence citizens’ views on polity formation in foreign policy. We now further explore the individual characteristics which are likely to impact these dynamics. The first characteristic we look at is ideology. While European foreign policy is not directly salient for national parties, party differences in stances on foreign policy, as well as views on European integration, should translate into partisan differences at the individual level. While right-wing voters are more likely than left-wing ones to support military spending and aggressive foreign policy (Wagner et al., 2017), they are less likely to support EU level defense policy (Schoen, 2008). Right-wing parties and their followers are primarily interested in promoting national interests in foreign policy and, therefore, have a preference to maintain sovereignty in that area (Raunio & Wagner, 2020). We therefore expect left-wing voters to be more in favor of both coordination and centralization than right-wing ones, and less in favor of national solutions, even in the short term.

- **H4a:** compared to the right, the left will have a higher preference for cooperation and then second for an integrated EU army. In other words, their intercepts are closer to *C* than *B*, but their coefficients are steeper in Figure 4.

Related to ideology is European identity. Moving foreign policy to the EU level, either through increased cooperation or centralization implies further EU integration and giving up of national sovereignty. Whether an individual has a stronger national or European identity affects their views on EU integration and the perceived legitimacy of which actor should be responsible for foreign policy (Hooghe & Marks, 2005). We therefore hypothesize that individuals with a stronger EU identity should be more supportive of both coordination and centralization, even in the short term. They should be less sensitive to the short-term costs implied by the J-curve and more concerned with the long-term benefits of further integration. Their slope on the J-curve should, therefore, be steeper upwards.

- **H4b:** respondents with strong EU identity will prefer an EU army over the other options because they are less sensitive to the short-term costs.

Lastly, we consider the heterogeneous effect of views on NATO. We expect individuals with different views on NATO to react differently to the US withdrawal from the conflict and from European security more broadly. Anti-NATO views are generally associated with lower concern for security issues. Such individuals are more likely to consider the downsides of NATO and of American military power. Pro-NATO views, on the other hand, are likely to be associated with individuals who are more concerned with security and see the US as a guarantor of European security. The latter group will, therefore, be more sensitive to the immediate costs of US withdrawal and have a strong preference to fill in the void. However, because the costs will be higher, their dip in the J-curve will be more pronounced, and will therefore show a larger gap between coordination and centralization, in favor of coordination, which could fill in the gap quicker. They should prefer both options to maintaining the status quo, or to national solutions.

- **H4c:** people with high trust in NATO will, in the short run, prefer more cooperation than centralization. In other words, their intercepts are closer to *C* than *B* in Figure 4;

We expect individual-level heterogeneities to aggregate up by country. Countries where elites and individuals are more NATO-skeptic (such as France and Italy) are more likely to prefer European security as an alternative to American security guarantees. They should, therefore, be less sensitive to the short-term costs of US withdrawal and more focused on the long term benefits. Conversely, pro-NATO countries (such as Poland) should be more sensitive to the short-term costs of US withdrawal and more likely to see EU security as a less efficient alternative of US security. Finland, at the time of writing not a member of NATO, has favorable views of the alliance (which it seeks to join) and should fall somewhere in between. Its embeddedness in the European security architecture should imply that respondents react to the security void generated by a US withdrawal, but less so than Poland, a NATO member with favorable views of the alliance.

The surprising implications of our approach is that, pace Tilly, Riker, Kelemen and McNamara, paradoxically, an external threat like the Russian invasion of Ukraine will put pressure on Europeans not necessarily to support polity centralization, but to further support an increase of the coordination among strong subunits in which they reside. The resulting process is that the impact of external crises on European polity formation is less “Tillian" than “Milwardian" (Milward, 1992), in the sense that coordination strengthens the subunits without necessarily centralizing the polity. This is due to the short time bias of external threats which make cooperation less costly than centralization. However, should the threat escalate and other security guarantees come under question, our theory predicts that in the long term, preferences would increase for centralizing the EU polity. Further coordination among member states in the short could also pave the way for long-term centralization. Our theory is, therefore, more optimistic about the long-term than the anti-bellicists (Genschel, 2022; Anghel & Jones, 2023).

## Data and Experimental Design

The data for this study was collected as part of a survey conducted in seven EU countries (Germany, France, Italy, Portugal, Poland, Hungary, and Finland) in the framework of the SOLID-ERC research project. Interviews were administered in July 2022 on national samples obtained using a quota design based on gender, age, macro-area of residence (NUTS-1), and education. The total sample size for the survey was 12,371, with national sample sizes varying between 1,024 and 2,084. Within this survey we fielded a factorial vignette experiment varying the two parameters influencing the J-curve as well as the potential capacity-building responses to these.

In our vignette experiment each survey respondent was presented with two policy scenarios in which various combinations of three factors were assigned. Factor 1 was dedicated to varying the slope condition and addressed the Russian actions and the level of threat they represent in as much a realistic manner as possible. Russian actions could stay constant (military efforts in Ukraine continue), could escalate and thus raise the saliency of the war (e.g., military efforts aimed at civilians), could spill-over into other, non-EU countries (Moldova), or into a European country (e.g., attacking a facility in Lithuania which is realistic given that Lithuania separates Belarus from Kaliningrad, and given that the Suwałki Gap-the shortest land route between Belarus and Kaliningrad-is one of NATO’s weak points in the East). Factor 2 aimed to address the security structure in which EU member states are embedded, part of both the intercept and slope conditions. Specifically, we varied the level of the United States security presence. Two scenarios are presented to the respondents in this respect. In one scenario, the United States continues to supply arms to Ukraine and maintains troops in EU countries. In the other, the United States announced a withdrawal of military support for Ukraine and the removal of a significant number of US troops from EU countries. It is worth noting here that in order to make sure respondents do not interpret this scenario as a consequence of a lower level of threat and, hence, a lower need for US presence, we specifically primed them to think of this scenario as a result of potential escalation and increase in threat. Finally, Factor 3 addressed the potential military policy responses to the combination of Factors 1 and 2. Four options were given to the respondents: maintaining the status quo, increasing national defense capacity independently at the Member State level, increasing military cooperation at the EU level, and building new centralized military capacity at the EU through the creation of an integrated EU army.^5^ Respondents had to evaluate the scenarios resulting from these combinations of factors on an 11-point scale. Table 1 presents this experimental design together with an example vignette presented to respondents. Appendix B lists all 32 possible combinations of our factors.

**Table 1:** Experimental design

Please read carefully the following hypothetical scenarios. The Russian government, on the war front, ...				
**Factor 1:**	**Level 1**	**Level 2**	**Level 3**	**Level 4**
Russian actions	is continuing its military efforts in Ukraine	is intensifying its military efforts against Ukrainian civilian targets (residential buildings, hospitals, civilians)	is extending its military campaign into Moldova, a non-EU member state	is attacking a military facility in Lithuania, an EU member state

The United States ...				
**Factor 2:**	**Level 1:**		**Level 2:**	
NATO actions	has announced it will continue supplying arms to Ukraine and maintaining troops in EU countries		fearing escalation, has announced a withdrawal of military support for Ukraine, and the removal of a significant number of US troops from EU countries.	

In response, ...				
**Factor 3:**	**Level 1**	**Level 2**	**Level 3**	**Level 4**
Policy response	EU member states maintain their current level of defense capacity	each EU member state increases their national defense capacity independently	EU member states agree to increase the defense capacity of the EU through greater military cooperation among themselves	EU member states agree to increase the defense capacity of the EU through the creation of an integrated EU army
**Example vignette:**	The Russian government is extending its military campaign into Moldova, a non-EU member state. The United States fearing escalation, has announced a withdrawal of military support for Ukraine, and the removal of a significant number of US troops from EU countries. In response, EU member states agree to increase the defense capacity of the EU through greater military cooperation among themselves.			

Our main goal is to estimate which characteristic of a scenario increases or decreases the appeal of that scenario, when varied independently of the other attributes included in the design, but also the interactions between these attributes. Since we are repeating measurements across respondents (two vignettes per respondent), the empirical strategy we adopt for analyzing this experiment relies on linear mixed-effect models, with 2-way and 3-way interactions, a random intercept for respondent, and country fixed effects. The full text of all questions together with their operationalization is presented in Appendix B, while descriptive statistics on these variables are shown in Appendix C, Figs. 22 and 23 and Tables 2 and 3.

## Results

**Fig. 5:**
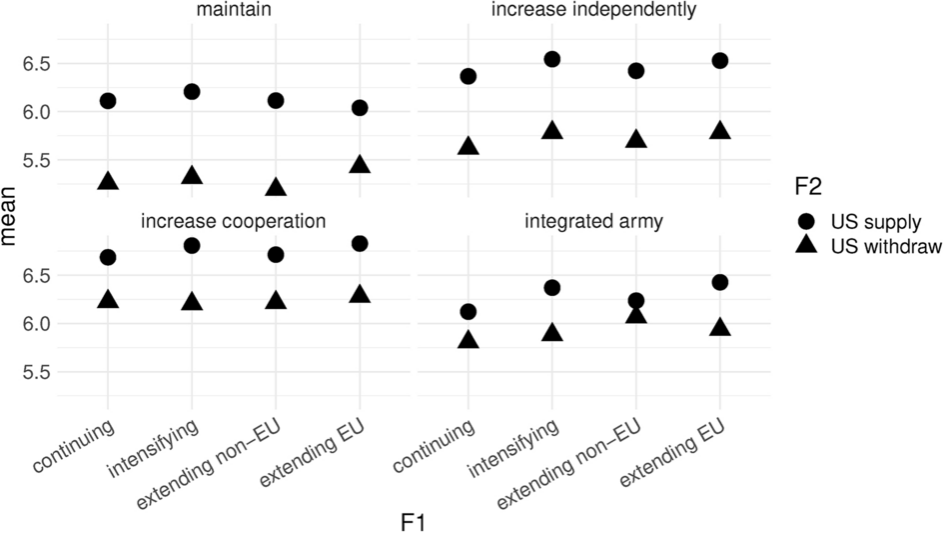
Descriptives

Figure 5 shows the predicted values across our three factors. On the y-axis we have our dependent variable, the predicted level of agreement of respondents with the policy action of EU member states. On the x-axis we have Factor 1, the increasing level of threat from Russia. Factor 2 is represented with shapes in the plot, circles representing the predicted values for when the US maintains commitment and presence, and triangles when it withdraws. Lastly, Factor 3 is represented in the breakdown of the 4 sub-figures. On the top left we see predicted values for when EU member states maintain the status quo of military capacity. On the top right EU member states increase their capacity independently at the national level. On the bottom left, EU member states increase military cooperation, and on the bottom right is the proposal for an integrated EU army.

**Fig. 6:**
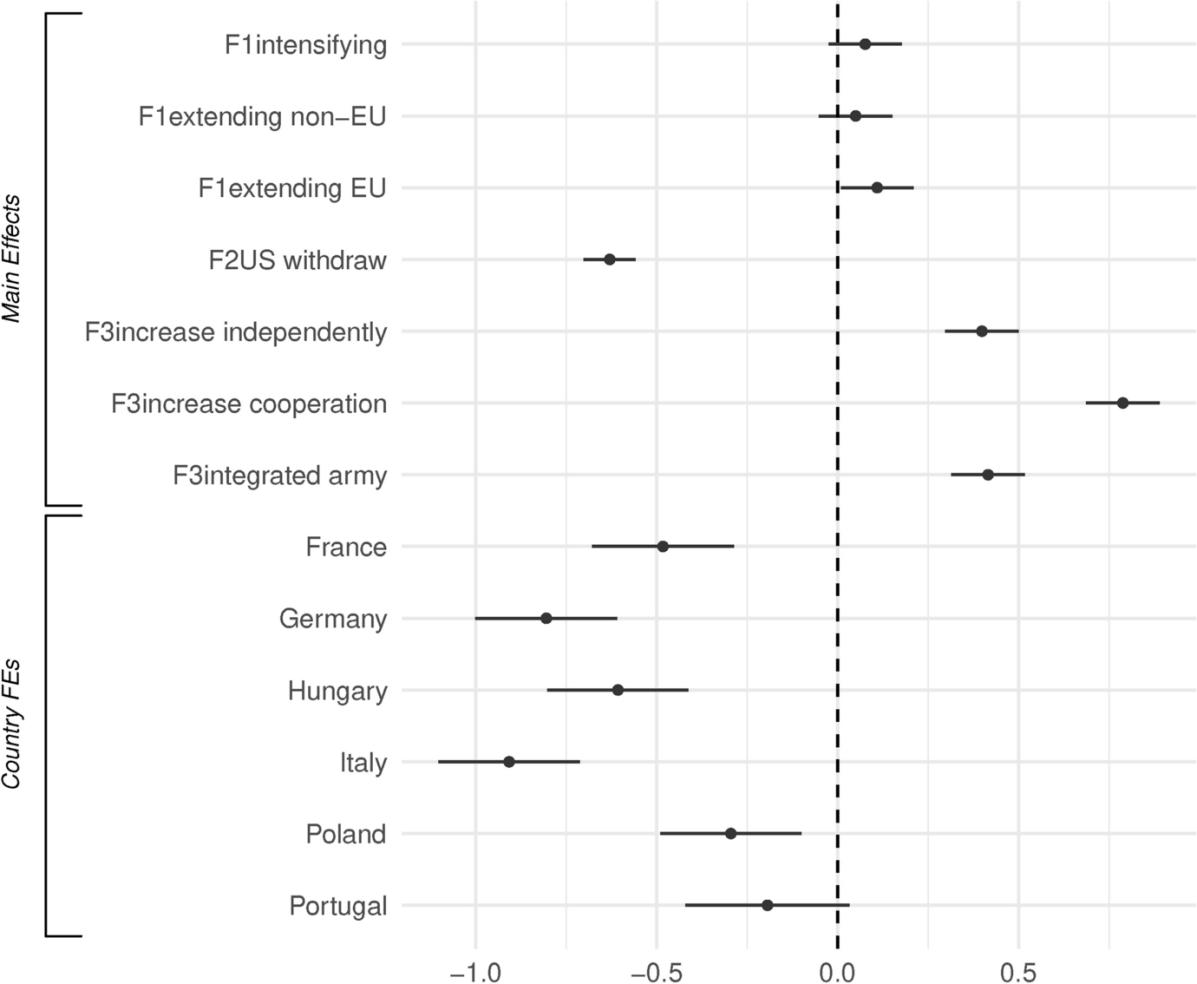
Main model

Several patterns already stand out. The first is that F1 does not seem to make a difference for respondents. That is, irrespective of how strong the escalation is by Russia, respondents have similar ratings to the actions of EU member states. The second is that respondents rate policy responses in scenarios where the US withdraws lower than when the US maintains a presence. Thirdly, and finally, we see that respondents, on average, seem to prefer increasing cooperation the most and maintaining existing policies the least. This provides initial support for H1. When comparing “increasing cooperation” and “integrated army” we note the lower rating of the latter. Since both scenarios imply increasing resources at the EU level, we attribute the lower rating of the “integrated army” to the short term costs associated with centralization during an acute crisis which requires a quick and effective response.^6^

We now dive deeper and further explore all of these dynamics by breaking down the effects. Figure 6 breaks down the main effects and the country-fixed effects. We can see here more clearly the negative effect of the US withdrawing, and the higher preference for increasing cooperation, while noting that both the integrated army and increasing national defense are preferred to the status quo.

**Fig. 7:**
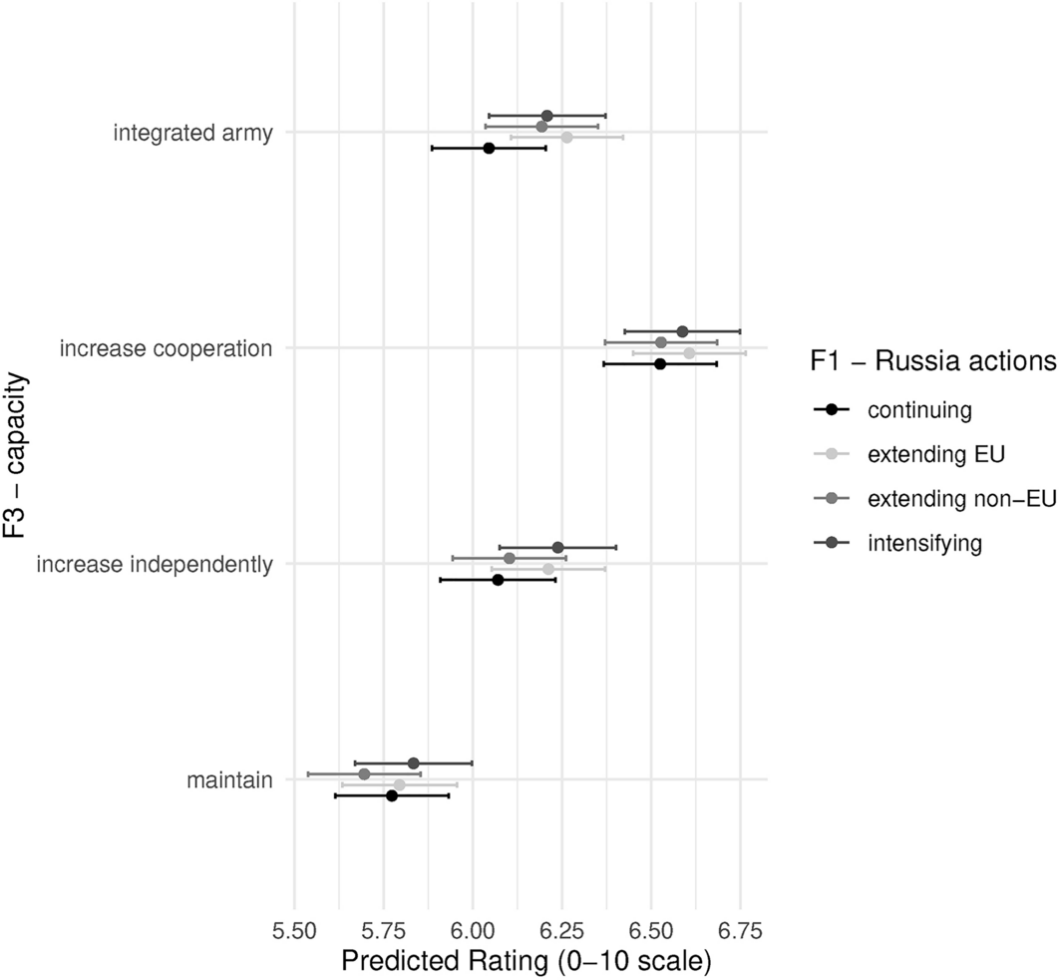
Interaction F1 - Russia’s action and F3 - defense capacity response - predicted

Figure 7 shows the predicted probabilities for the interaction between F1 and F3 (for marginal effects, see Fig. 17 in the Appendix). We see that irrespective of Russia’s actions, respondents on average prefer increasing cooperation, and rate maintaining the status quo the lowest (reference category). Do these results imply that respondents are indifferent to the war? We believe not. Respondents clearly prefer policies that increase the military capacity of member states. It does seem, however, that respondents are not sensitive to escalation in the war when it comes to its effects on military policies. This is the short-term bias stressed in our theoretical framework. Respondents are currently in the lower part of the J, the initial period where higher threat requires immediate action, which is more efficient with existing capacity. In the short run, major policy reforms are considered costly, especially under threat. Hence, the escalation of the existing threat does little to respondents’ existing preferences.

We note that our treatment may be under-representing the true effect of a real-world Russian escalation. Respondents might not fully internalize such an escalation of threat under a hypothetical scenario as compared to real life, where, among other factors, the media and political elites would react and influence their perceptions. Nonetheless, there is evidence to suggest that this null effect is specific to defense policy. We have conducted an experiment with a similar factor, that varied the same condition on Russian escalation and its effects on preference for energy sanctions and energy policy at the EU and member state level. As Fig. 16 in the Appendix shows, this manipulation had large effect sizes and interacted significantly with energy policy preferences. This gives us further grounds to believe that the null effect for defense policy has to do with the specifics of the policy field, in this case the short-term costs of higher centralization in the face of an immediate threat, which requires immediate action. To our surprise, this logic also seems to hold for coordination. Thus, we find mixed results for H3: as expected, higher threat does not increase preferences for centralization, however, contrary to expectations, it also does not increase preferences for coordination.

**Fig. 8:**
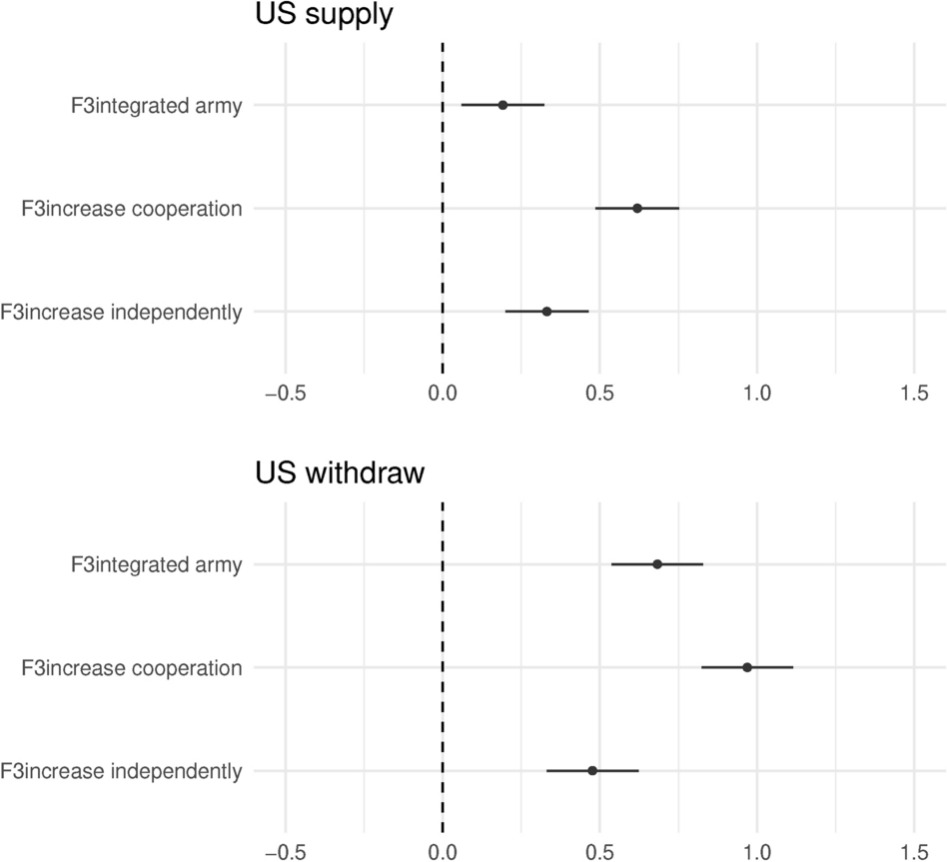
Interaction F2 - US’s action and F3 - defense capacity response

We now turn to the effect of the US withdrawing. Figure 8 shows the marginal effects of our Factor 3, the policy response, by levels of US actions, on top, the US maintaining support and presence, and on the bottom, the US withdrawing. What we see is that the marginal effect of policies of Factor 3 increases when the US withdraws. In other words, in the absence of US protection, the difference between maintaining the status quo and all other policies becomes much larger. Respondents prefer increased cooperation overall but all three options have stronger effects compared to the status quo. Thus, following our predictions, respondents prefer increased cooperation in the face of a security vacuum. Cooperation remains the most efficient policy in the short term, whereas capacity building would require time in order to reap benefits.

**Fig. 9:**
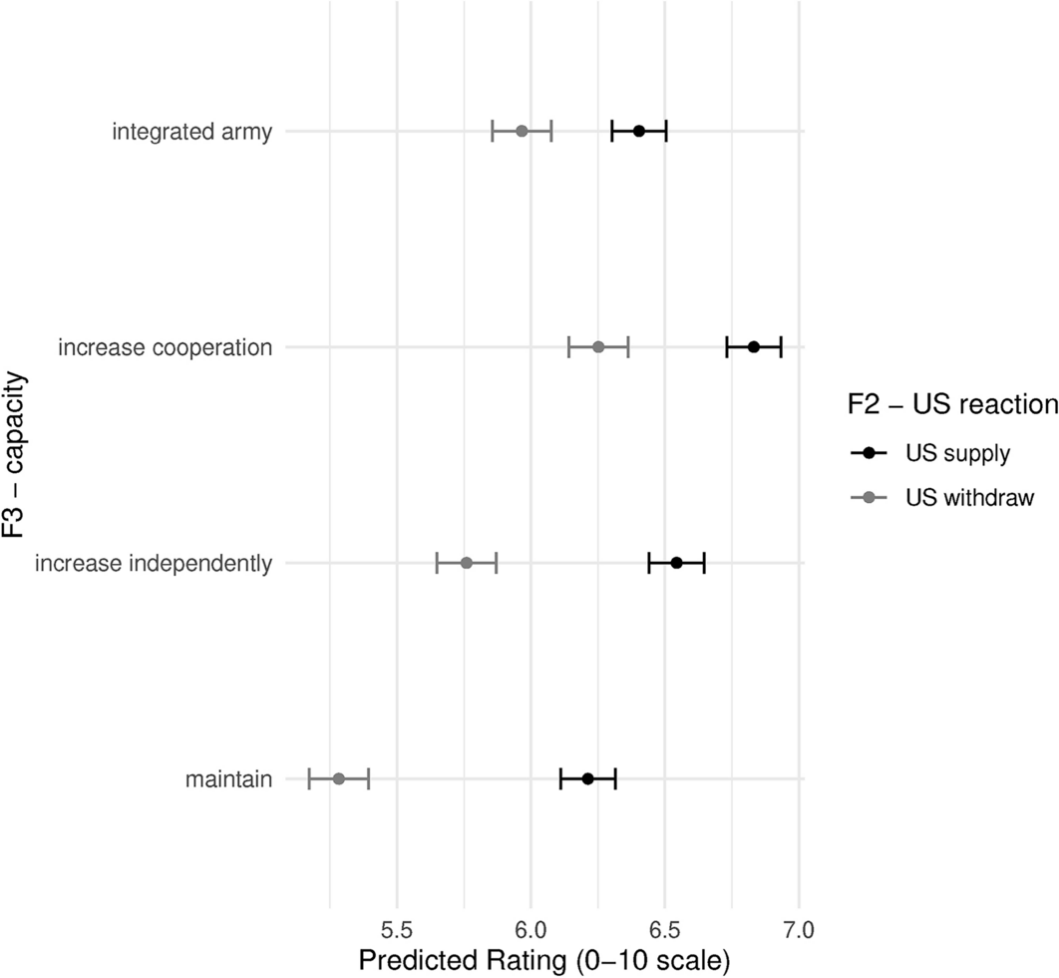
Interaction F2 - US’s action and F3 - defense capacity response - predicted

At the same time, we see that the overall effect of US withdrawal is negative. Figure 9 shows the predicted values for the interaction. What we see is that across different types of EU member state actions, in scenarios where the US withdraws, respondents report lower ratings. We interpret the overall effect and the previously discussed marginal effect as evidence for a J-curve of policy rating. Simply put, under our scenarios, in the short term, respondents are choosing the “best of the worst options”. That is to say, in the short term, US withdrawal would be catastrophic for the Western efforts in Ukraine. There is no good policy under that scenario. Therefore respondents rate them lower. Nonetheless, within these policy options, respondents take the “best of the worst” by rating cooperation, integrated EU army, and independent increase in capacity much higher. In the short term, all possible policies, in the absence of the US, are less desired in respondents’ eyes. This gives us an indication that respondents find the most effective policies to be when the US and the EU work together, rather than apart (Wang & Moise, 2023). We thus find support for our H2: in the short term, respondents react to the security vacuum by rating all policies lower but prefer coordination to a wider degree than in the status quo with US security guarantees.

Overall, our experimental findings show evidence for a J-curve in demand for EU-level security. In the short term, EU-level policies might be ineffective because of the void left by the US withdrawal. However, we see that within this scenario respondents are more eager to opt for EU-level solutions, as those options are rated much higher. At the macro level, we believe this suggests that the EU should develop its security structure already. The possible consequences of a US withdrawal (not impossible given domestic US politics and the possibility of a second Trump term) could be catastrophic. The EU needs to preempt the J-curve.

**Fig. 10:**
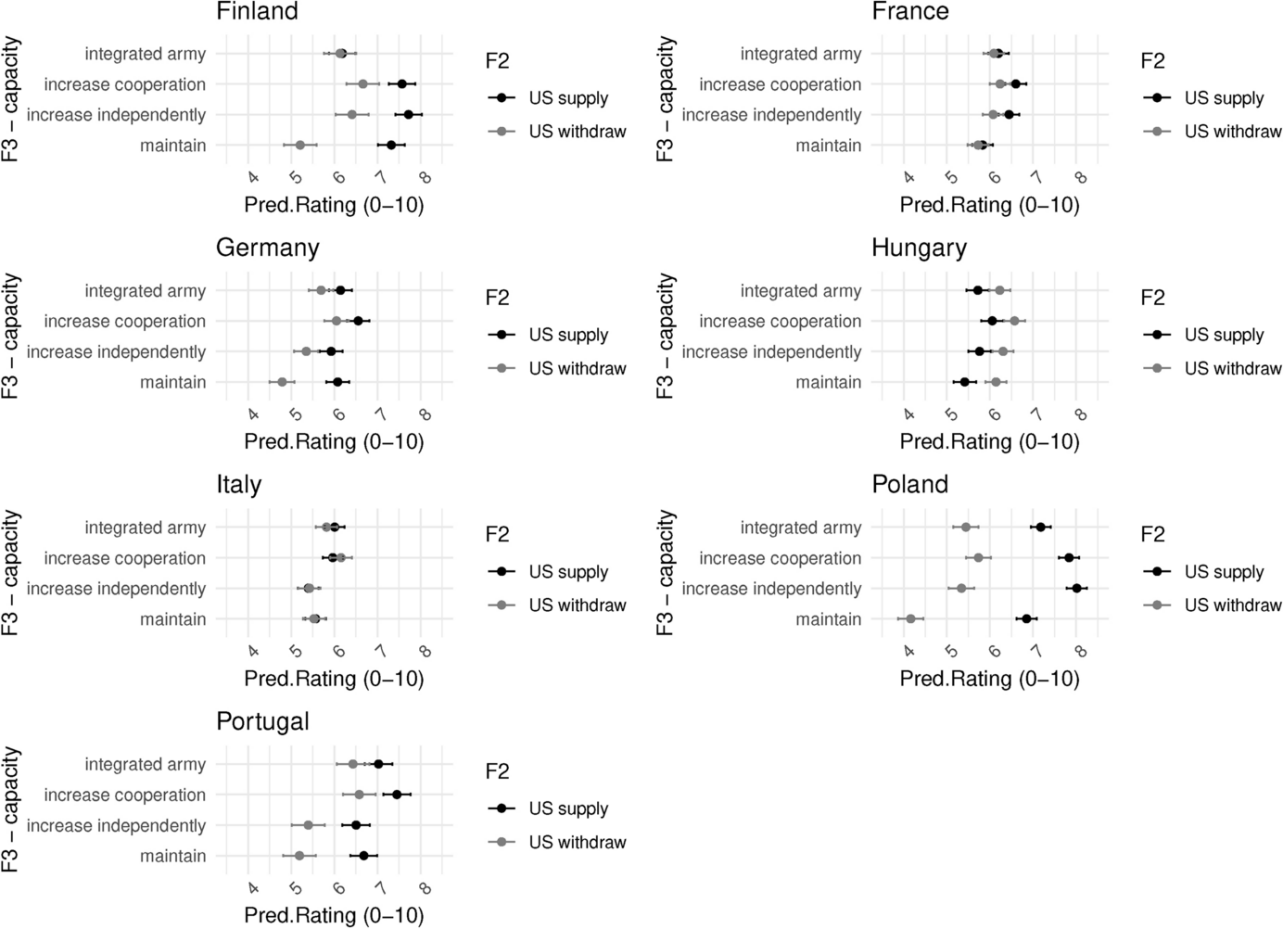
Interaction F2 - US’s action and F3 - defense capacity response - by country predicted

We see further evidence for this interpretation in Fig. 10. In Poland, the most pro-NATO and pro-Ukraine country, respondents view US withdrawal as particularly catastrophic in the short term. Italians, much more NATO-skeptic, are indifferent. Finland also shows the most pronounced effect of US withdrawal on differential policy preferences. With the US maintaining security guarantees in the region, Finns rate centralization the lowest, below the status quo. In the scenario of a US withdrawal, Finns rate centralization on par with coordination and national solutions, and much higher than maintaining the status quo.

**Fig. 11:**
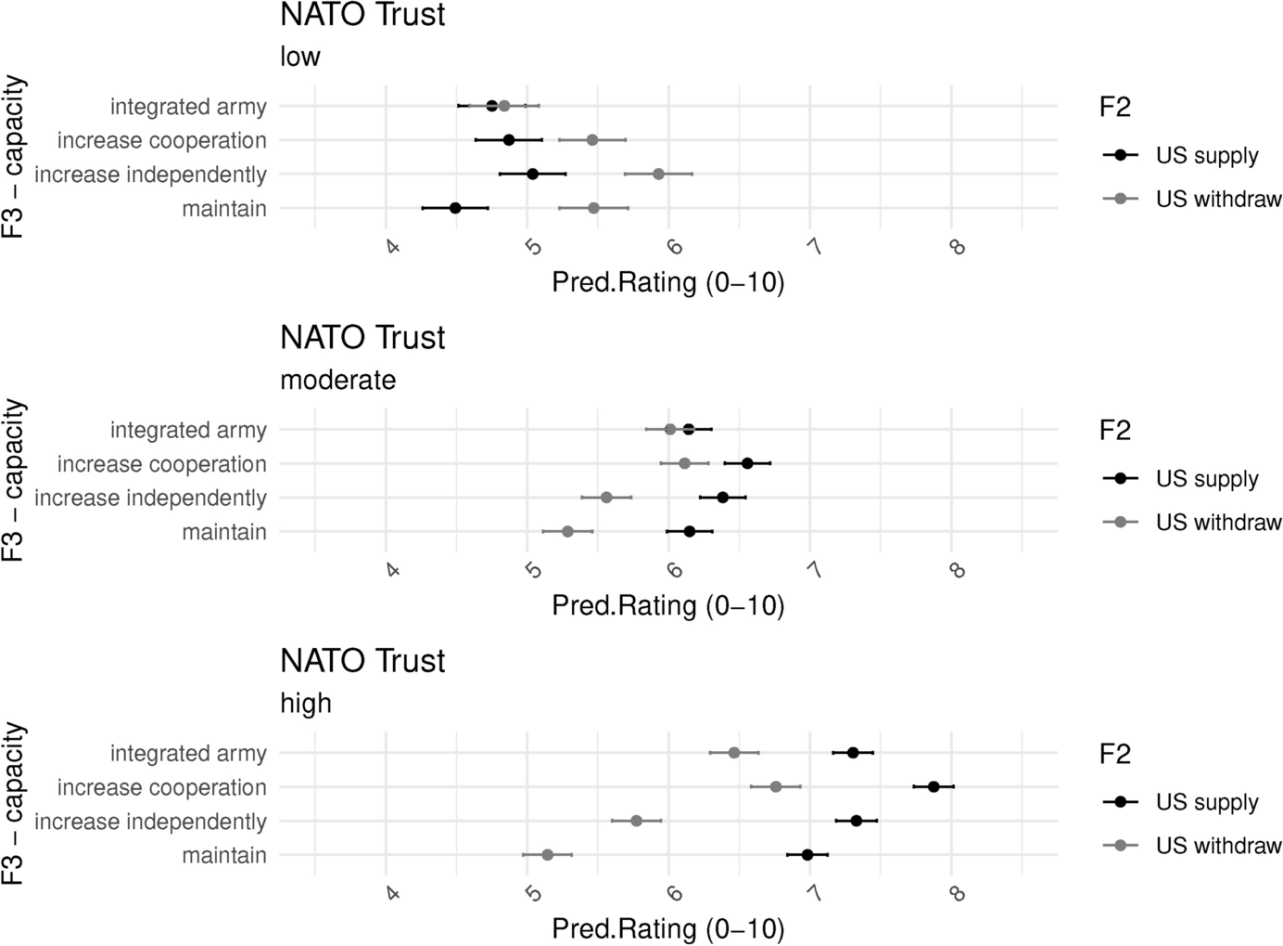
Interaction F2 - US’s action and F3 - defense capacity response - by trust in NATO predicted

We test this effect more directly in Fig. 11, which confirms our suspicion. Respondents who trust NATO (bottom) rate scenarios of US withdrawal much more negatively. They prefer both US and EU security protection. The gap between maintaining the status quo and increasing capacity, particularly in the form of increased cooperation, increases as the US withdraws. On the other hand, respondents who do not trust NATO welcome US withdrawal even in the short term. We thus find support for H4c.

**Fig. 12:**
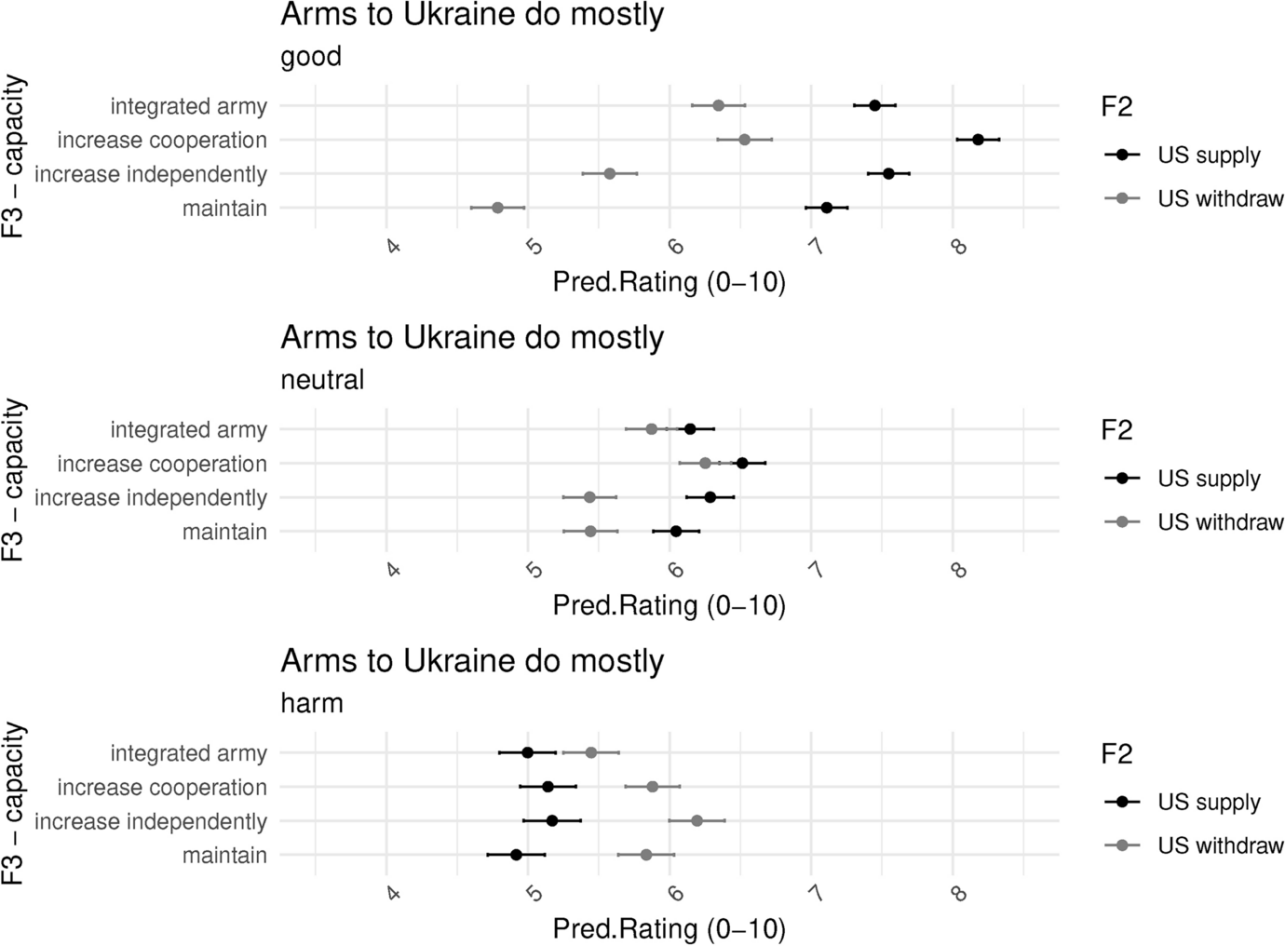
Interaction F2 - US’s action and F3 - defense capacity response - by arms to Ukraine doing harm/good

We further corroborate this finding by looking at interactions with three other factors. Figure 12 shows the interaction with our indicator for pacifism, whether respondents think that sending arms to Ukraine does more harm than good. Respondents who are more pacifist rate scenarios with the US withdrawing higher. They also show little difference between policy options. In fact, under a scenario of the US withdrawing, they rate the option of the EU integrated army lower than the status quo.

**Fig. 13:**
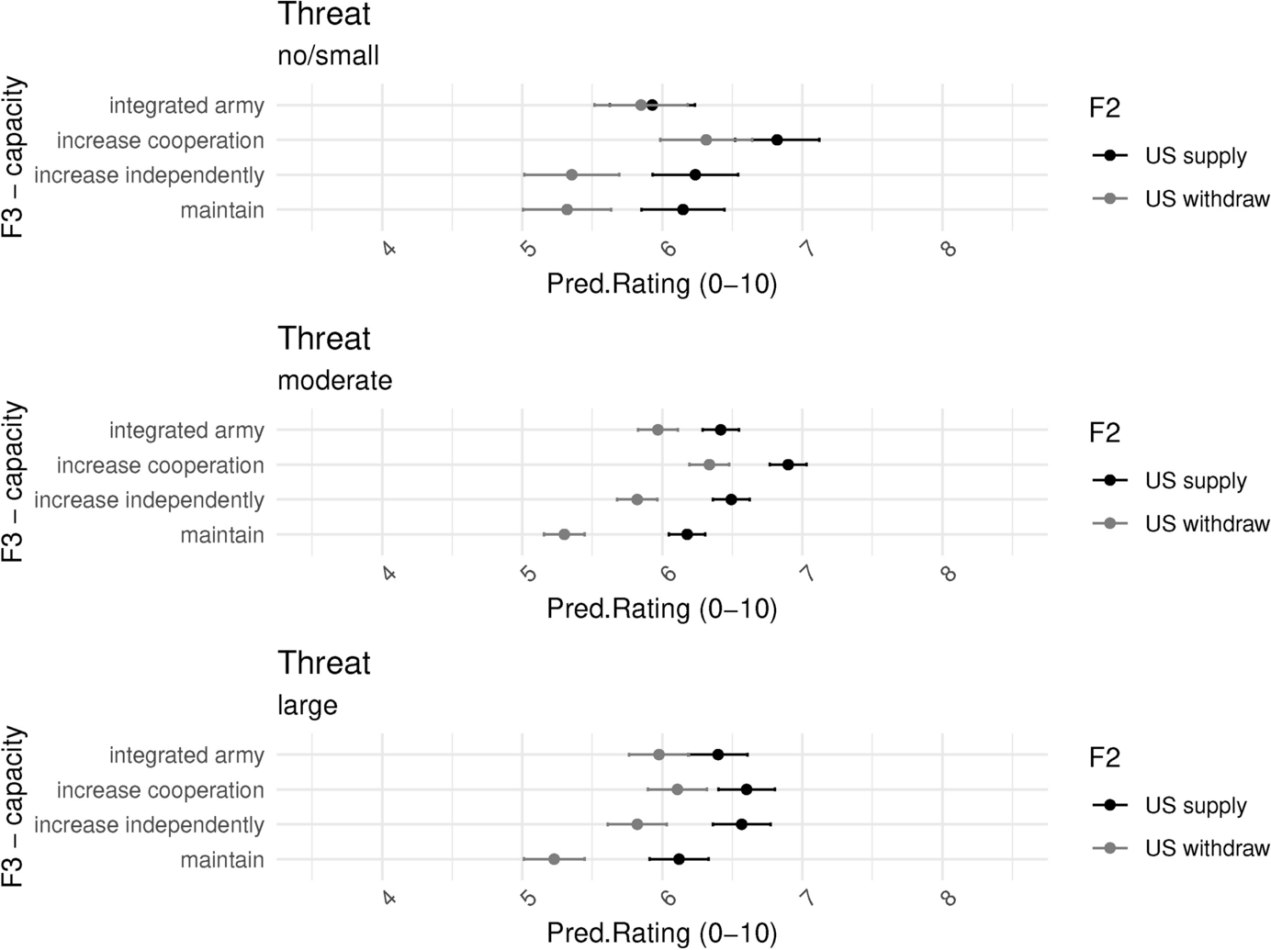
Interaction F2 - US’s action and F3 - defense capacity response - by threat perception of the war

Finally, we look at an alternative explanation, namely, whether threat perception moderates this effect. Figure 13 shows the effect by levels of threat perception of the war. The findings corroborate our interpretation of insensitivity of respondents to threat, for defense policy. Higher perceived threat does not, on average, influence the rating of policies. It seems that respondents are insensitive to growing or existing threat from the war when it comes to defense. We also see that it does not interact with the withdrawal of the US. It appears that US withdrawal is connected to perceptions of NATO and Russia and not threat.

**Fig. 14:**
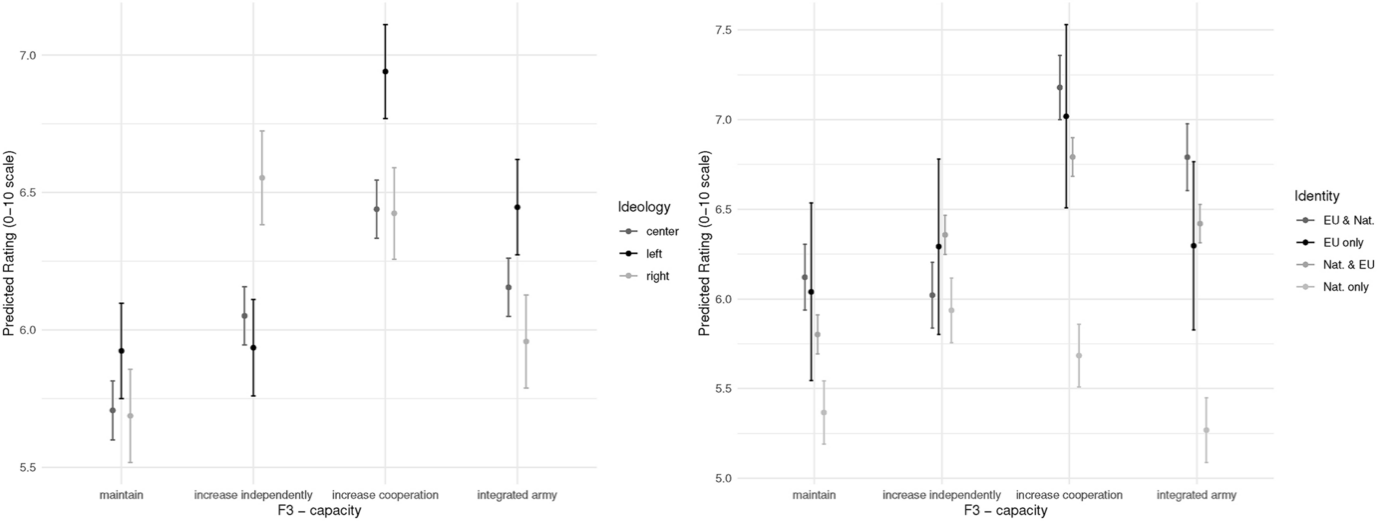
F3 - defense capacity response - by ideology and identity

**Fig. 16:**
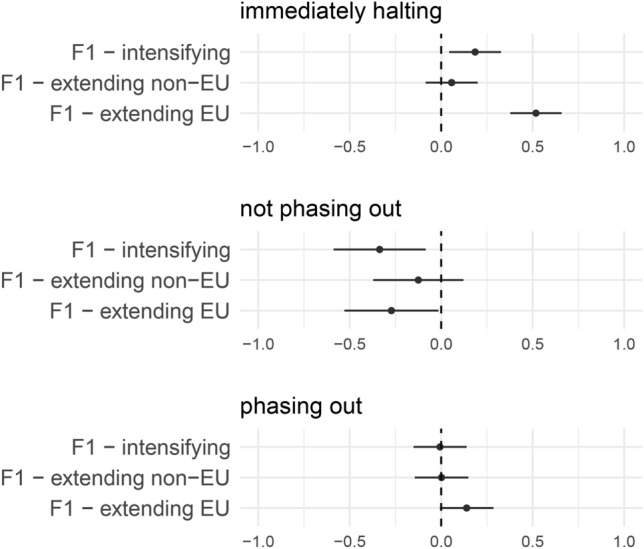
The effect of escalation on energy policy preferences

**Fig. 17:**
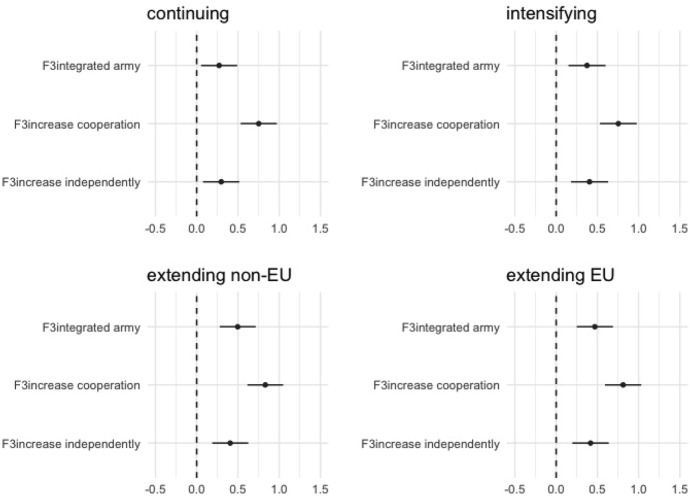
Interaction F1 - Russia’s action and F3 - defense capacity response

**Fig. 22:**
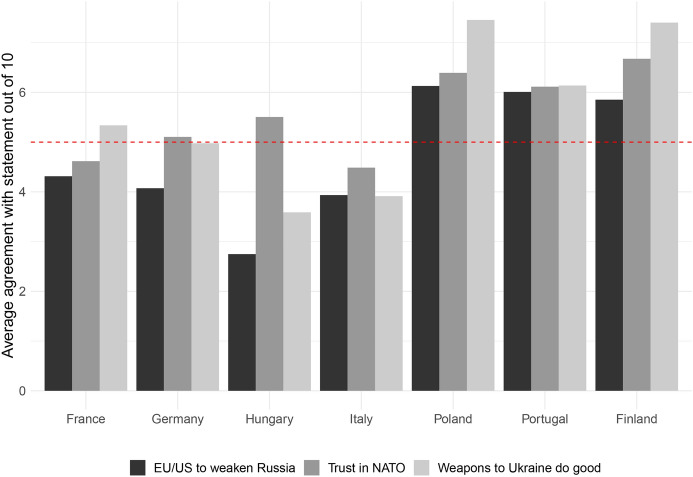
Country averages for questions on trust in NATO, weakeining Russia, and arms to UA. Note: The scale of “Weapons to Ukraine” was reversed for ease of interpretation. Higher values indicate average agreement that weapons to more good than harm. See original wording in Appendix B.

**Fig. 23:**
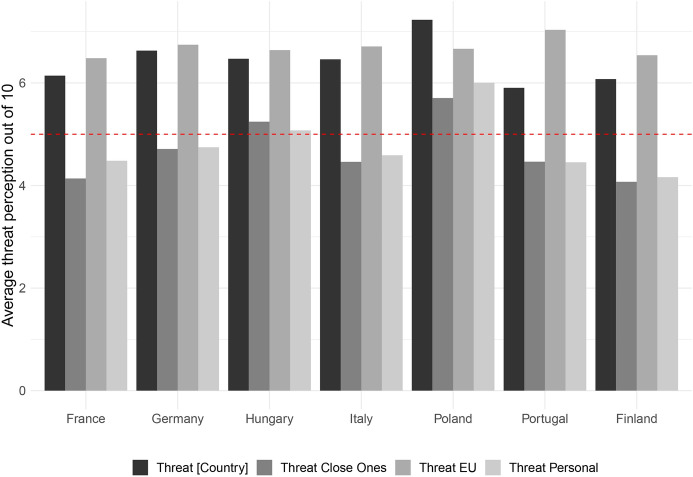
Country averages for questions on Threat

Finally, we turn to heterogeneous effects by ideology and identity. Figure 14 shows our expected effect for left-right. We see no difference when it comes to maintaining the status quo. However, all other policies show some degree of ideological polarization. The left and right wing agree that the status quo is inferior but they do not agree on the solution. Left-wing voters prefer increasing cooperation and an integrated EU army, while right-wing voters prefer increasing national capacity (more than left-wing respondents) and increased cooperation (less than left-wing respondents). We believe this is driven by the GAL-TAN dimension of left-right, as more cosmopolitan left-wing voters prefer EU options, while more nationally-oriented right-wing voters prefer the nation-state. When we compare the “increased cooperation“ and “army” scenarios (both of which imply more resources at the EU level) with the national solution, we see that right wing respondents are not less in favor of EU-level resources through cooperation, but are against the loss of sovereignty implied by a unified army. European identity (same figure, right panel) corroborates this interpretation. Indeed, those who identify primarily with their national identity only, are far less likely to prefer increasing cooperation at the EU level, or an EU-level army. While all groups prefer increased cooperation to an EU army, due to short-term costs, it is only right-wing and nationalist respondents that also see a “sovereignty” cost, in addition.^7^ We thus find support for H4a and H4b. This finding has important implications for the postfunctionalism literature. While we see differences between the groups, it is important to note that even right-wing individuals support increased cooperation at the EU level compared to the status quo, at about the same level as national solutions. This implies that politicians have greater leeway in pursuing integration and do not face the possibility of imminent backlash from their voters.

## Discussion and Conclusion

Our paper contributes to the discussion on how crises shape the formation of the European polity. Many commentators wish that crises would strengthen Europe, push it onto the federalist path, and reinforce its strategic autonomy. We look at the most likely case for polity building in the area of defense, namely a common external threat. Our argument and evidence suggest that in *the short-run*, people support a different pathway than the implicit calls for further federalization, supranationalization, and centralization. We argue theoretically that preferences for polity centralization in the face of an external threat follow a J-curve, with increased preferences in the short-term for utilizing more efficient national and coordination mechanisms, compared to centralization. We tested the short term implications of our theory by looking at the effect of two conditions, which we called the intercept and the slope of polity preferences.

The intercept, the initial policy preferences at the start of an external threat, is shaped by the structure of the polity (weak versus strong units) and external security guarantees. We show that EU citizens, who are part of a polity with strong sub-units and with strong external security guarantees, favor coordination over polity centralization. Greater coordination can also be utilized as an engine for further EU integration in the long term.

Our experimental design allowed us to test two factors that impacted the slope of policy preferences, showing the paradoxical effects of increased threat. First, we analyzed the impact of the US withdrawing from the conflict, thus diminishing EU security guarantees. We saw that the short-term security vacuum left by the US actually decreased support for policies across the board, highlighting that respondents are aware of the grave consequences of such a scenario. Within the set of policies, respondents increased their support for coordination and, to a lesser degree, for an EU army. Secondly, we looked at the varying levels of Russian escalation in the war. We saw that irrespective of the escalation scenario (including attacking an EU member state), respondents did not significantly alter their policy preferences. Greater escalation does not seem to spur demand for polity centralization. Our theory suggests that this is in part due to the short-term costs of new policies in dealing with imminent threats.

Our paper has several limitations, offering promising avenues for further research. First, we focus primarily on the demand side of politics, namely, voter preferences. Further research can investigate the degree to which the supply side, namely politicians, react to voter preferences. Second, due to methodological constraints, Factor 3 had to limit the number of options available for the policy response. New surveys could further explore whether enhanced coordination through NATO rather than the EU would be the preferred option for respondents.

Thirdly, our study only permits us to study the short-run implications of threats on polity formation. Our theoretical framework and findings lead us to hypothesize a growing demand for centralization in the long run. Further research can explicitly test this hypothesis by, for instance, fielding a similar experiment several years into the Russian war in Ukraine. Recent developments in European defense policy, as well as actions by EU leaders and member states, point to the plausibility of a trend of greater coordination and even centralization in the long term. EU leaders have called for subsidizing defense production at the EU level,^8^ and aim to produce at least half of the block’s military equipment domestically by 2035.^9^ Nordic countries have centralized their military air fleet into a unified command.^10^ Following Trump’s comments about abandoning NATO allies, we also see further calls by EU leaders to strengthen European defense and integration.^11^

Fourthly, further research can investigate these dynamics in different policy fields and for different crises. A within-crisis, static, analysis can explore the J-curve dynamic for different policy fields. Our theory predicts that in policy fields where member states have more capacity compared to the EU, and where member state interests diverge, the intercept drops (intercept condition closer to C than B) and the short-term bias intensifies. Conversely, in fields with weak state capacity, and where there are high potential collective benefits, the intercept rises (intercept condition closer to A than B) and centralization is more likely even in the short term. Contrast for instance the refugee and the COVID crises. In the former, member states have most of the capacity and disagreed on the policy response, and are thus reluctant to centralize (Kriesi et al., 2021; Kriesi et al. 2024; Eilstrup-Sangiovanni 2021, for extensive treatments of the reluctance to centralize and national rebordering). In the latter, overwhelmed states capacities in health and fiscal policies led to increased solidarity and preferences for EU centralization on the demand side (Oana & Truchlewski, 2024) and more policy centralization in the form of common vaccine procurement and a stabilization fund (Kriesi et al., 2024; Truchlewski et al., 2025). Similarly to the case of defense, where the collective good is provided externally by the US, in the refugee crisis the EU managed to externalize the policy solution through the EU-Turkey agreement on refugees.

Furthermore, a between-crisis, dynamic analysis can explore the J-curve dynamic for different crises and test the effect of cumulative crises. The intercept of the J-curve can be modified by each successive crisis as crisis solutions create a new equilibrium. For instance, if successful, Next Generation EU (NGEU, created during the COVID-19 pandemic) can move the intercept towards 0 as countries see that pooling common resources to decrease external shocks actually works, which lays the groundwork for further solidarity instruments.

Our theory, therefore, has important implications for current debates on European polity building. When it comes to the demand side, it suggests that the bellicists are too optimistic about polity centralization in the event of an external threat. However, it also suggests that the anti-bellicists are too pessimistic. The long-term may yet bring polity-building, through coordination if not direct centralization, in a Milwardian rather than Tillian process. Scenarios in which US security guarantees come into doubt (even partially), may further exacerbate EU polity-building in defense.

## Figures and Tables

**Fig. 15:**
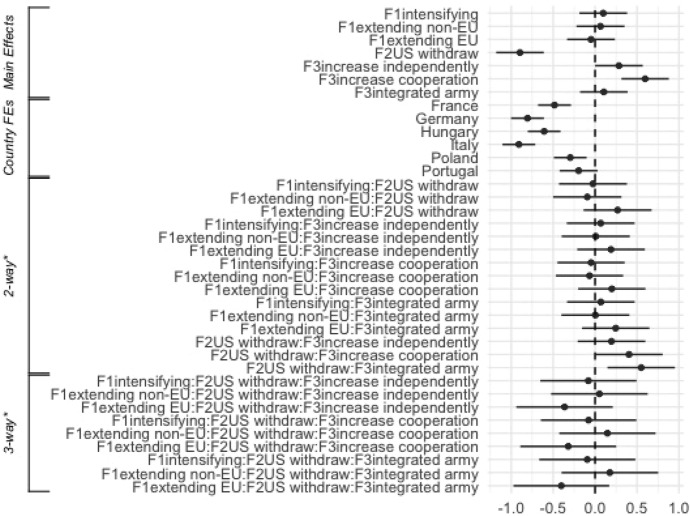
Model with all interactions

**Fig. 18:**
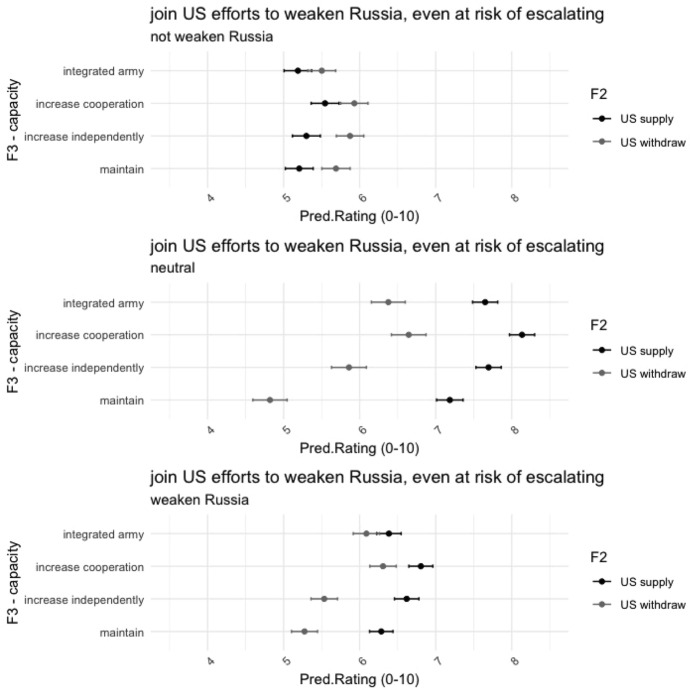
Interaction F2 - US’s action and F3 - defense capacity response - by continuing to help military efforts at the risk of escalation

**Fig. 19:**
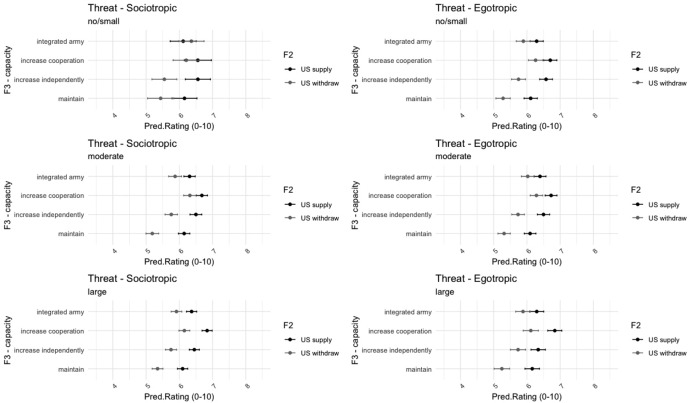
Interaction F2 - US’s action and F3 - defense capacity response - by threat perception of the war - egotropic and sociotropic

**Fig. 20:**
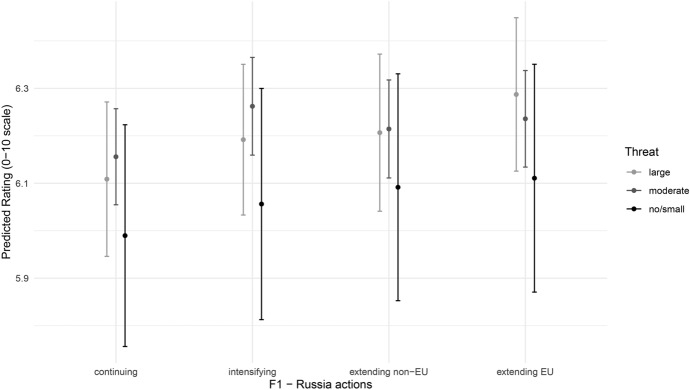
Interaction between F1 - Russia’s actions and threat perceptions

**Fig. 21:**
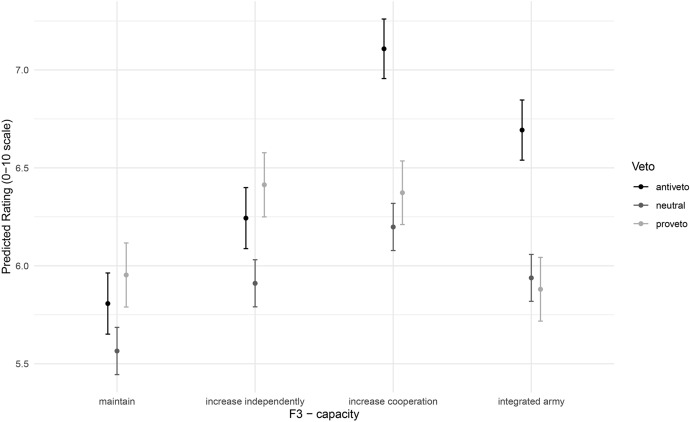
Interaction between F3 - Policy Response and Support for National-level Veto

**Fig. 24:**
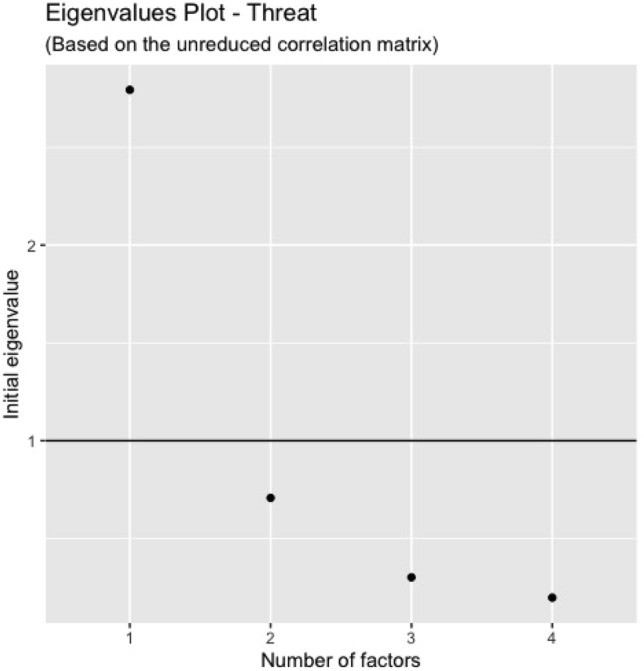
Eigenvalues plot

**Fig. 25:**
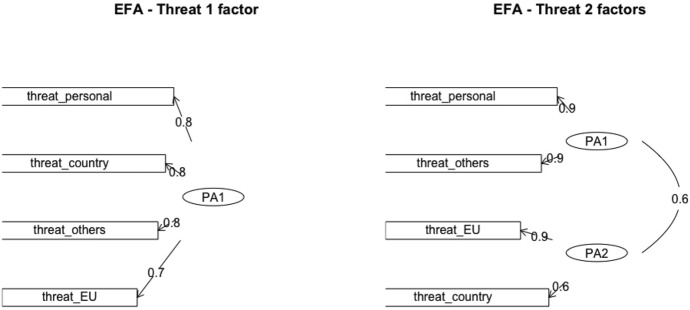
Exploratory factor analysis plot

**Fig. 26:**
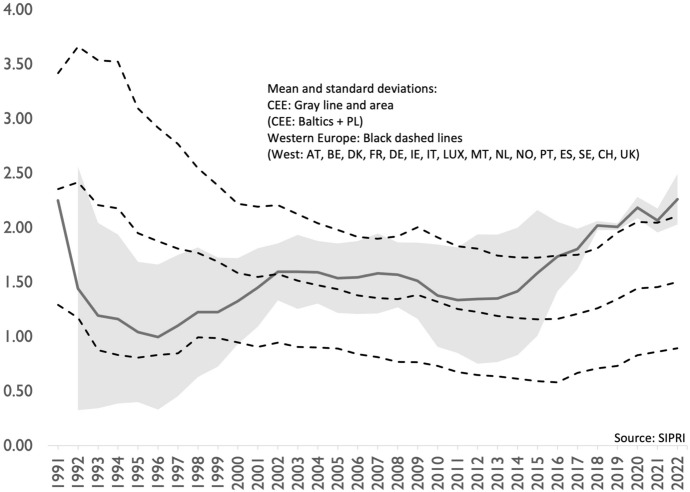
Defense Spending in Western Countries and Countries Bordering Russia

**Fig. 27:**
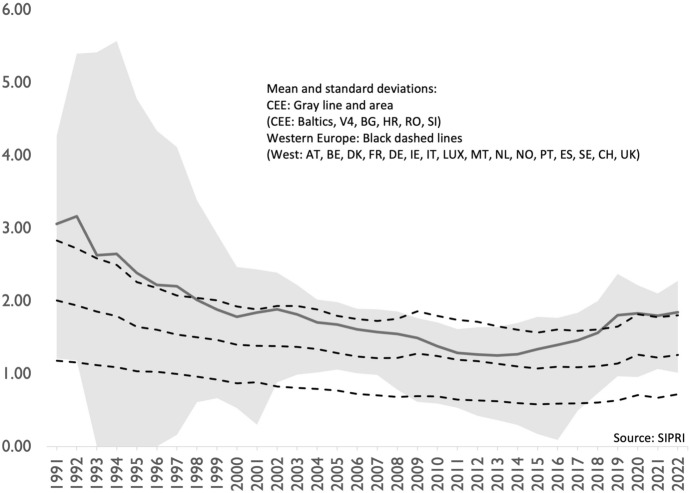
Defense Spending in Western and Postcommunist Countries

## Data Availability

Replication data and code are available at https://doi.org/10.7910/DVN/QUVPD7

## A Additional Figures

See Appendix Figs. 15, 16, 17, 18, 19, 20 and 21.

## B Full Question Wording

### B.1 All factorial vignette combinations

1. 1/1/1 The Russian government is continuing its military efforts in Ukraine. The United States has announced it will continue supplying arms to Ukraine and maintaining troops in EU countries. In response, EU member states maintain their current level of defense capacity.
2. 1/1/2 The Russian government is continuing its military efforts in Ukraine. The United States has announced it will continue supplying arms to Ukraine and maintaining troops in EU countries. In response, each EU member state increases their national defense capacity independently.
3. 1/1/3 The Russian government is continuing its military efforts in Ukraine. The United States has announced it will continue supplying arms to Ukraine and maintaining troops in EU countries. In response, EU member states agree to increase the defense capacity of the EU through greater military cooperation among themselves.
4. 1/1/4 The Russian government is continuing its military efforts in Ukraine. The United States has announced it will continue supplying arms to Ukraine and maintaining troops in EU countries. In response, EU member states agree to increase the defense capacity of the EU through the creation of an integrated EU army.
5. 1/2/1 The Russian government is continuing its military efforts in Ukraine. The United States, fearing escalation, has announced a withdrawal of military support for Ukraine, and the removal of a significant number of US troops from EU. In response, EU member states maintain their current level of defense capacity.
6. 1/2/2 The Russian government is continuing its military efforts in Ukraine. The United States, fearing escalation, has announced a withdrawal of military support for Ukraine, and the removal of a significant number of US troops from EU. In response, each EU member state increases their national defense capacity independently.
7. 1/2/3 The Russian government is continuing its military efforts in Ukraine. The United States, fearing escalation, has announced a withdrawal of military support for Ukraine, and the removal of a significant number of US troops from EU. In response, EU member states agree to increase the defense capacity of the EU through greater military cooperation among themselves.
8. 1/2/4 The Russian government is continuing its military efforts in Ukraine. The United States, fearing escalation, has announced a withdrawal of military support for Ukraine, and the removal of a significant number of US troops from EU. In response, EU member states agree to increase the defense capacity of the EU through the creation of an integrated EU army.
9. 2/1/1 The Russian government is intensifying its military efforts against Ukrainian civilian targets (residential buildings, hospitals, civilians). The United States has announced it will continue supplying arms to Ukraine and maintaining troops in EU countries. In response, EU member states maintain their current level of defense capacity.
10. 2/1/2 The Russian government is intensifying its military efforts against Ukrainian civilian targets (residential buildings, hospitals, civilians). The United States has announced it will continue supplying arms to Ukraine and maintaining troops in EU countries. In response, each EU member state increases their national defense capacity independently
11. 2/1/3 The Russian government is intensifying its military efforts against Ukrainian civilian targets (residential buildings, hospitals, civilians). The United States has announced it will continue supplying arms to Ukraine and maintaining troops in EU countries. In response, EU member states agree to increase the defense capacity of the EU through greater military cooperation among themselves.
12. 2/1/4 The Russian government is intensifying its military efforts against Ukrainian civilian targets (residential buildings, hospitals, civilians). The United States has announced it will continue supplying arms to Ukraine and maintaining troops in EU countries. In response, EU member states agree to increase the defense capacity of the EU through the creation of an integrated EU army.
13. 2/2/1 The Russian government is intensifying its military efforts against Ukrainian civilian targets (residential buildings, hospitals, civilians). The United States, fearing escalation, has announced a withdrawal of military support for Ukraine, and the removal of a significant number of US troops from EU. In response, EU member states maintain their current level of defense capacity.
14. 2/2/2 The Russian government is intensifying its military efforts against Ukrainian civilian targets (residential buildings, hospitals, civilians). The United States, fearing escalation, has announced a withdrawal of military support for Ukraine, and the removal of a significant number of US troops from EU. In response,each EU member state increases their national defense capacity independently.
15. 2/2/3 The Russian government is intensifying its military efforts against Ukrainian civilian targets (residential buildings, hospitals, civilians). The United States, fearing escalation, has announced a withdrawal of military support for Ukraine, and the removal of a significant number of US troops from EU. In response, EU member states agree to increase the defense capacity of the EU through greater military cooperation among themselves.
16. 2/2/4 The Russian government is intensifying its military efforts against Ukrainian civilian targets (residential buildings, hospitals, civilians). The United States, fearing escalation, has announced a withdrawal of military support for Ukraine, and the removal of a significant number of US troops from EU. In response, EU member states agree to increase the defense capacity of the EU through the creation of an integrated EU army.
17. 3/1/1 The Russian government is extending its military campaign into Moldova, a non-EU member state. The United States has announced it will continue supplying arms to Ukraine and maintaining troops in EU countries. In response, EU member states maintain their current level of defense capacity.
18. 3/1/2 The Russian government is extending its military campaign into Moldova, a non-EU member state. The United States has announced it will continue supplying arms to Ukraine and maintaining troops in EU countries. In response, each EU member state increases their national defense capacity independently.
19. 3/1/3 The Russian government is extending its military campaign into Moldova, a non-EU member state. The United States has announced it will continue supplying arms to Ukraine and maintaining troops in EU countries. In response, EU member states agree to increase the defense capacity of the EU through greater military cooperation among themselves.
20. 3/1/4 The Russian government is extending its military campaign into Moldova, a non-EU member state. The United States has announced it will continue supplying arms to Ukraine and maintaining troops in EU countries. In response, EU member states agree to increase the defense capacity of the EU through the creation of an integrated EU army.
21. 3/2/1 The Russian government is extending its military campaign into Moldova, a non-EU member state. The United States, fearing escalation, has announced a withdrawal of military support for Ukraine, and the removal of a significant number of US troops from EU. In response, EU member states maintain their current level of defense capacity.
22. 3/2/2 The Russian government is extending its military campaign into Moldova, a non-EU member state. The United States, fearing escalation, has announced a withdrawal of military support for Ukraine, and the removal of a significant number of US troops from EU. In response, each EU member state increases their national defense capacity independently.
23. 3/2/3 The Russian government is extending its military campaign into Moldova, a non-EU member state. The United States, fearing escalation, has announced a withdrawal of military support for Ukraine, and the removal of a significant number of US troops from EU. In response, EU member states agree to increase the defense capacity of the EU through greater military cooperation among themselves.
24. 3/2/4 The Russian government is extending its military campaign into Moldova, a non-EU member state. The United States, fearing escalation, has announced a withdrawal of military support for Ukraine, and the removal of a significant number of US troops from EU. In response, EU member states agree to increase the defense capacity of the EU through the creation of an integrated EU army.
25. 4/1/1 The Russian government is attacking a military facility in Lithuania, an EU member state. The United States has announced it will continue supplying arms to Ukraine and maintaining troops in EU countries. In response, EU member states maintain their current level of defense capacity.
26. 4/1/2 The Russian government is attacking a military facility in Lithuania, an EU member state. The United States has announced it will continue supplying arms to Ukraine and maintaining troops in EU countries. In response, each EU member state increases their national defense capacity independently.
27. 4/1/3 The Russian government is attacking a military facility in Lithuania, an EU member state. The United States has announced it will continue supplying arms to Ukraine and maintaining troops in EU countries. In response, EU member states agree to increase the defense capacity of the EU through greater military cooperation among themselves.
28. 4/1/4 The Russian government is attacking a military facility in Lithuania, an EU member state. The United States has announced it will continue supplying arms to Ukraine and maintaining troops in EU countries. In response, EU member states agree to increase the defense capacity of the EU through the creation of an integrated EU army.
29. 4/2/1 The Russian government is attacking a military facility in Lithuania, an EU member state. The United States, fearing escalation, has announced a withdrawal of military support for Ukraine, and the removal of a significant number of US troops from EU. In response, EU member states maintain their current level of defense capacity.
30. 4/2/2 The Russian government is attacking a military facility in Lithuania, an EU member state. The United States, fearing escalation, has announced a withdrawal of military support for Ukraine, and the removal of a significant number of US troops from EU. In response, each EU member state increases their national defense capacity independently.
31. 4/2/3 The Russian government is attacking a military facility in Lithuania, an EU member state. The United States, fearing escalation, has announced a withdrawal of military support for Ukraine, and the removal of a significant number of US troops from EU. In response, EU member states agree to increase the defense capacity of the EU through greater military cooperation among themselves.
32. 4/2/4 The Russian government is attacking a military facility in Lithuania, an EU member state. The United States, fearing escalation, has announced a withdrawal of military support for Ukraine, and the removal of a significant number of US troops from EU. In response, EU member states agree to increase the defense capacity of the EU through the creation of an integrated EU army.

### B.2 Question wording

- **Energy Experiment - Figure** 16 The Russian government, on the war front,...
  1. is continuing its military efforts in Ukraine
  2. is intensifying its military efforts against Ukrainian civilian targets (residential buildings, hospitals, civilians)
  3. is extending its military campaign into Moldova, a non-EU member state
  4. is attacking a military facility in Lithuania, an EU member state.
    Russian military efforts are financed through energy exports. In the meantime, EU member states are considering a common policy of ...
- 1. immediately halting Russian energy imports within months, which may result in loss of jobs and industry
  2. phasing out Russian energy imports slowly, which will make energy more expensive in the medium term
  3. NOT phasing out of Russian energy imports.
  ...
  1. The costs of this policy will be covered by individuals and businesses consuming energy
  2. The costs of this policy will be covered by each national government, even if this might mean higher taxes in proportion to national energy dependence
  3. The costs of this policy will be shared among all EU member states equally, even if this might mean higher taxes irrespective of national energy dependence
  4. BLANK (for Level 3 of Factor 2)
    Example vignette: Please read carefully the following hypothetical scenario. The Russian government, on the war front, is extending its military campaign into Moldova, a non-EU member state. Russian military efforts are financed through energy exports. In the meantime, EU member states are considering a common policy of phasing out Russian energy imports slowly, which will make energy more expensive in the medium term. The costs of this policy will be covered by individuals and businesses consuming energy. Given this scenario, to what extent do you approve or disapprove of the considered policy? (0-Completely disapprove to 10-Completely approve)
- **Ideology:**
  In political matters, people sometimes talk of “left” and “right” . How would you place your views on this scale, where 0 means “Left” and 10 means “Right”?
- **Trust in NATO:**
  Generally speaking, how much do you personally trust each of the following institutions? ... NATO (0-Completely distrust to 10-Completely trust)
- **Arms to Ukraine:**
  Sending weapons to Ukraine does more harm than good. (0-Completely disagree to 10-Completely agree)
- **US efforts to weaken Russia:**
  EU countries should join the US efforts to weaken Russia militarily, even at the risk of escalating the war. (0-Completely disagree to 10-Completely agree)
- **Threat Perception Questions:**
  How much of a threat, if any, does the Ukraine conflict pose for...
  - The safety and stability of [COUNTRY]?
  - The safety and stability of the European Union?
  - Your own, personal safety?
  - The safety of people you know personally?
  Select a value from 0 to 10, where 0 means “No threat at all" and 10 means “A major threat".
- **EU Identity:**
  Do you see yourself as”...?
  - European Only
  - European and (Nationality)
  - (Nationality) and European
  - (Nationality) only
  - None of these
- **Member state veto, Figure** 21:
  There is now a debate about whether the EU’s foreign and security policy should be developed. How much do you agree or disagree with the following?
  Single member states can now block EU foreign policy decisions, such as sanctions against third countries. This rule should be maintained.
  Select a value from 0 to 10, where 0 means “Disagree completely" and 10 means “Agree completely”.

## C Descriptive Figures and Tables

See Appendix Figs. 22, 23 and Tables 2, 3.

**Table 2:** Summary descriptives table by country: France, Germany, Italy, and Portugal

	France	Germany	Italy	Portugal
	N=1924	N=1947	N=1972	N=962
Trust in NATO	4.62 (2.87)	5.10 (3.02)	4.49 (2.93)	6.11 (2.58)
Weapons to Ukraine do harm	4.66 (3.45)	5.02 (3.69)	6.09 (3.31)	3.86 (3.35)
EU join US weaken Russia	4.31 (3.27)	4.07 (3.39)	3.94 (3.28)	6.01 (3.28)
Threat to Country	6.14 (2.61)	6.63 (2.60)	6.46 (2.61)	5.90 (2.61)
Threat to EU	6.48 (2.49)	6.75 (2.50)	6.71 (2.55)	7.03 (2.47)
Threat to self	4.48 (3.02)	4.75 (3.04)	4.59 (3.04)	4.45 (3.00)
Threat to close ones	4.14 (3.13)	4.71 (3.11)	4.46 (3.16)	4.46 (2.91)
Ideology:				
Right	685 (42.8%)	474 (26.7%)	654 (41.8%)	307 (37.7%)
Left	519 (32.5%)	672 (37.8%)	622 (39.7%)	281 (34.5%)
Center	395 (24.7%)	630 (35.5%)	290 (18.5%)	226 (27.8%)
EU Identity:				
European only	38 (1.98%)	100 (5.34%)	56 (3.08%)	35 (3.68%)
European and $QNATIONALITY	279 (14.5%)	363 (19.4%)	310 (17.1%)	143 (15.1%)
$QNATIONALITY and European	922 (47.9%)	891 (47.5%)	952 (52.4%)	588 (61.9%)
$QNATIONALITY only	685 (35.6%)	520 (27.7%)	500 (27.5%)	184 (19.4%)

**Table 3:** Summary descriptives table by country: Hungary, Poland, and Finland

	Hungary	Poland	Finland
	N=1971	N=1938	N=1015
Trust in NATO	5.50 (3.03)	6.39 (2.69)	6.67 (2.54)
Weapons to Ukraine do harm	6.41 (3.74)	2.54 (3.21)	2.60 (2.92)
EU join US weaken Russia	2.75 (3.14)	6.13 (3.37)	5.85 (2.95)
Threat to Country	6.47 (2.84)	7.23 (2.51)	6.08 (2.45)
Threat to EU	6.64 (2.73)	6.67 (2.57)	6.54 (2.32)
Threat to self	5.08 (3.13)	6.01 (2.97)	4.16 (2.84)
Threat to close ones	5.24 (3.09)	5.71 (3.05)	4.07 (2.88)
Ideology:			
Right	609 (36.1%)	568 (34.2%)	395 (48.1%)
Left	492 (29.1%)	606 (36.5%)	285 (34.7%)
Center	588 (34.8%)	487 (29.3%)	141 (17.2%)
EU Identity:			
European only	77 (3.91%)	111 (5.81%)	9 (0.89%)
European and $ QNATIONALITY	465 (23.6%)	411 (21.5%)	130 (12.8%)
$ QNATIONALITY and European	1031 (52.3%)	1080 (56.5%)	593 (58.4%)
$ QNATIONALITY only	398 (20.2%)	308 (16.1%)	283 (27.9%)

## D Exploratory factor analysis for the threat variable

We used principal axis factoring with oblimin rotation using the psych package in R. Figure 24 shows that there is only one factor with eigenvalues higher than 1, confirming our selection of a single factors as optimal. However, the one-factor model presented sub-optimal model fit statistics (TLI = 0.509), while the two-factor model had optimal fit statistics (TLI = 1.012). Figure 25 shows item loadings on the factors. Given this, as a robustness check we decided to run results using both. For this, we simply averaged over all four variables or over pairs of two variables. As shown, the results do not change by using a different aggregation.

## E Tables corresponding to the plotted figures

See Appendix Tables 4, 5, 6, 7, 8, 9, 10, 11, 12, 13.

**Table 4:** Table for main model in Fig 6

	*Dependent variable:*
	RATING
F1intensifying	0.076
	(0.051)
F1extending non-EU	0.050
	(0.052)
F1extending EU	$$0.109^{**}$$
	(0.051)
F2US withdraw	$$-0.630^{***}$$
	(0.037)
F3increase independently	$$0.399^{***}$$
	(0.052)
F3increase cooperation	$$0.789^{***}$$
	(0.052)
F3integrated army	$$0.416^{***}$$
	(0.052)
as.factor(Country)France	$$-0.483^{***}$$
	(0.100)
as.factor(Country)Germany	$$-0.805^{***}$$
	(0.100)
as.factor(Country)Hungary	$$-0.607^{***}$$
	(0.099)
as.factor(Country)Italy	$$-0.908^{***}$$
	(0.100)
as.factor(Country)Poland	$$-0.295^{***}$$
	(0.100)
as.factor(Country)Portugal	$-0.194^{*}$
	(0.116)
Constant	$$6.479^{***}$$
	(0.094)
Observations	20,428
Log Likelihood	−49,716.760
Akaike Inf. Crit.	99,465.520
Bayesian Inf. Crit.	99,592.320

Note: $$^{*}$$p<0.1; $$^{**}$$p<0.05; $$^{***}$$p<0.01

**Table 5:** Table corresponding to Fig 7

	F3	predicted	std.error	conf.low	conf.high	F1
1	maintain	5.773	0.081	5.615	5.931	continuing
2	increase independently	6.070	0.082	5.909	6.231	continuing
3	increase cooperation	6.525	0.081	6.366	6.683	continuing
4	integrated army	6.045	0.081	5.886	6.204	continuing
5	maintain	5.833	0.083	5.670	5.997	intensifying
6	increase independently	6.238	0.083	6.075	6.401	intensifying
7	increase cooperation	6.587	0.082	6.426	6.749	intensifying
8	integrated army	6.208	0.083	6.045	6.371	intensifying
9	maintain	5.696	0.081	5.538	5.854	extending non-EU
10	increase independently	6.102	0.081	5.944	6.261	extending non-EU
11	increase cooperation	6.527	0.080	6.370	6.684	extending non-EU
12	integrated army	6.193	0.080	6.035	6.351	extending non-EU
13	maintain	5.794	0.082	5.634	5.955	extending EU
14	increase independently	6.212	0.081	6.053	6.370	extending EU
15	increase cooperation	6.606	0.080	6.449	6.764	extending EU
16	integrated army	6.264	0.080	6.106	6.421	extending EU

**Table 6:** Table corresponding to Fig 9

	F3	predicted	std.error	conf.low	conf.high	F2
1	maintain	6.212	0.052	6.111	6.314	US supply
2	increase independently	6.544	0.052	6.442	6.646	US supply
3	increase cooperation	6.832	0.052	6.730	6.933	US supply
4	integrated army	6.404	0.052	6.302	6.505	US supply
5	maintain	5.283	0.057	5.172	5.394	US withdraw
6	increase independently	5.760	0.056	5.649	5.870	US withdraw
7	increase cooperation	6.252	0.056	6.142	6.362	US withdraw
8	integrated army	5.966	0.056	5.856	6.076	US withdraw

**Table 7:** Table corresponding to Fig 10

	F3	predicted	std.error	conf.low	conf.high	country	F2
1	maintain	7.321	0.157	7.013	7.629	FI	US supply
2	increase independently	7.720	0.156	7.414	8.026	FI	US supply
3	increase cooperation	7.570	0.156	7.263	7.876	FI	US supply
4	integrated army	6.185	0.156	5.880	6.490	FI	US supply
5	maintain	5.206	0.192	4.829	5.584	FI	US withdraw
6	increase independently	6.410	0.194	6.029	6.791	FI	US withdraw
7	increase cooperation	6.664	0.193	6.285	7.042	FI	US withdraw
8	integrated army	6.131	0.190	5.758	6.504	FI	US withdraw
9	maintain	5.830	0.123	5.588	6.072	FR	US supply
10	increase independently	6.445	0.122	6.205	6.685	FR	US supply
11	increase cooperation	6.601	0.123	6.359	6.842	FR	US supply
12	integrated army	6.199	0.123	5.959	6.440	FR	US supply
13	maintain	5.725	0.125	5.480	5.971	FR	US withdraw
14	increase independently	6.075	0.122	5.837	6.314	FR	US withdraw
15	increase cooperation	6.237	0.121	6.000	6.473	FR	US withdraw
16	integrated army	6.091	0.123	5.849	6.333	FR	US withdraw
17	maintain	6.075	0.136	5.808	6.343	GM	US supply
18	increase independently	5.926	0.135	5.662	6.190	GM	US supply
19	increase cooperation	6.553	0.134	6.290	6.817	GM	US supply
20	integrated army	6.142	0.136	5.875	6.409	GM	US supply
21	maintain	4.788	0.147	4.500	5.076	GM	US withdraw
22	increase independently	5.347	0.143	5.067	5.627	GM	US withdraw
23	increase cooperation	6.050	0.145	5.765	6.334	GM	US withdraw
24	integrated army	5.691	0.146	5.406	5.977	GM	US withdraw
25	maintain	5.416	0.133	5.155	5.676	HU	US supply
26	increase independently	5.759	0.133	5.498	6.021	HU	US supply
27	increase cooperation	6.055	0.131	5.799	6.311	HU	US supply
28	integrated army	5.720	0.133	5.460	5.980	HU	US supply
29	maintain	6.141	0.125	5.896	6.386	HU	US withdraw
30	increase independently	6.304	0.125	6.060	6.549	HU	US withdraw
31	increase cooperation	6.571	0.123	6.330	6.812	HU	US withdraw
32	integrated army	6.228	0.124	5.984	6.472	HU	US withdraw
33	maintain	5.564	0.121	5.326	5.801	IT	US supply
34	increase independently	5.396	0.121	5.159	5.632	IT	US supply
35	increase cooperation	5.961	0.119	5.727	6.196	IT	US supply

**Table 8:** Table corresponding to Fig 10 continued

	F3	predicted	std.error	conf.low	conf.high	country	F2
36	integrated army	6.006	0.118	5.775	6.237	IT	US supply
37	maintain	5.525	0.131	5.269	5.781	IT	US withdraw
38	increase independently	5.424	0.131	5.168	5.680	IT	US withdraw
39	increase cooperation	6.154	0.130	5.899	6.409	IT	US withdraw
40	integrated army	5.817	0.128	5.567	6.067	IT	US withdraw
41	maintain	6.851	0.118	6.619	7.083	PL	US supply
42	increase independently	8.017	0.120	7.782	8.253	PL	US supply
43	increase cooperation	7.837	0.119	7.604	8.070	PL	US supply
44	integrated army	7.179	0.118	6.948	7.409	PL	US supply
45	maintain	4.163	0.149	3.872	4.455	PL	US withdraw
46	increase independently	5.341	0.152	5.042	5.639	PL	US withdraw
47	increase cooperation	5.733	0.147	5.445	6.022	PL	US withdraw
48	integrated army	5.443	0.149	5.151	5.736	PL	US withdraw
49	maintain	6.683	0.160	6.370	6.996	PT	US supply
50	increase independently	6.503	0.165	6.181	6.826	PT	US supply
51	increase cooperation	7.451	0.162	7.134	7.767	PT	US supply
52	integrated army	7.024	0.163	6.704	7.344	PT	US supply
53	maintain	5.190	0.193	4.811	5.569	PT	US withdraw
54	increase independently	5.395	0.194	5.013	5.776	PT	US withdraw
55	increase cooperation	6.577	0.194	6.196	6.957	PT	US withdraw
56	integrated army	6.431	0.193	6.052	6.809	PT	US withdraw

**Table 9:** Table corresponding to Fig 11

	F3	predicted	std.error	conf.low	conf.high	NATO trust	F2
1	maintain	4.492	0.118	4.261	4.723	low	US supply
2	increase independently	5.039	0.118	4.808	5.270	low	US supply
3	increase cooperation	4.871	0.119	4.637	5.104	low	US supply
4	integrated army	4.751	0.120	4.515	4.987	low	US supply
5	maintain	5.471	0.124	5.229	5.713	low	US withdraw
6	increase independently	5.930	0.121	5.694	6.166	low	US withdraw
7	increase cooperation	5.461	0.119	5.228	5.695	low	US withdraw
8	integrated army	4.838	0.125	4.594	5.082	low	US withdraw
9	maintain	6.149	0.082	5.988	6.310	moderate	US supply
10	increase independently	6.384	0.082	6.224	6.544	moderate	US supply
11	increase cooperation	6.558	0.081	6.399	6.717	moderate	US supply
12	integrated army	6.142	0.082	5.983	6.302	moderate	US supply
13	maintain	5.286	0.089	5.111	5.462	moderate	US withdraw
14	increase independently	5.561	0.088	5.388	5.734	moderate	US withdraw
15	increase cooperation	6.114	0.086	5.945	6.283	moderate	US withdraw
16	integrated army	6.012	0.087	5.841	6.182	moderate	US withdraw
17	maintain	6.983	0.073	6.840	7.125	high	US supply
18	increase independently	7.329	0.073	7.185	7.472	high	US supply
19	increase cooperation	7.876	0.072	7.734	8.017	high	US supply
20	integrated army	7.305	0.072	7.164	7.446	high	US supply
21	maintain	5.144	0.087	4.973	5.315	high	US withdraw
22	increase independently	5.773	0.088	5.600	5.946	high	US withdraw
23	increase cooperation	6.759	0.089	6.585	6.934	high	US withdraw
24	integrated army	6.465	0.087	6.294	6.635	high	US withdraw

**Table 10:** Table corresponding to Fig 12

	F3	predicted	std.error	conf.low	conf.high	Arms supply	F2
1	maintain	7.110	0.075	6.963	7.257	good	US supply
2	increase independently	7.547	0.074	7.402	7.692	good	US supply
3	increase cooperation	8.179	0.076	8.031	8.327	good	US supply
4	integrated army	7.450	0.074	7.304	7.595	good	US supply
5	maintain	4.786	0.095	4.600	4.972	good	US withdraw
6	increase independently	5.578	0.097	5.387	5.768	good	US withdraw
7	increase cooperation	6.530	0.097	6.340	6.720	good	US withdraw
8	integrated army	6.346	0.096	6.158	6.533	good	US withdraw
9	maintain	6.046	0.082	5.884	6.207	meutral	US supply
10	increase independently	6.287	0.085	6.121	6.453	meutral	US supply
11	increase cooperation	6.514	0.082	6.353	6.674	meutral	US supply
12	integrated army	6.145	0.085	5.979	6.312	meutral	US supply
13	maintain	5.442	0.097	5.252	5.631	meutral	US withdraw
14	increase independently	5.436	0.094	5.251	5.621	meutral	US withdraw
15	increase cooperation	6.252	0.093	6.070	6.434	meutral	US withdraw
16	integrated army	5.873	0.092	5.691	6.054	meutral	US withdraw
17	maintain	4.917	0.103	4.715	5.119	harm	US supply
18	increase independently	5.171	0.103	4.970	5.372	harm	US supply
19	increase cooperation	5.141	0.100	4.944	5.338	harm	US supply
20	integrated army	4.997	0.101	4.799	5.195	harm	US supply
21	maintain	5.835	0.100	5.638	6.032	harm	US withdraw
22	increase independently	6.194	0.099	5.999	6.389	harm	US withdraw
23	increase cooperation	5.880	0.097	5.689	6.070	harm	US withdraw
24	integrated army	5.446	0.100	5.251	5.642	harm	US withdraw

**Table 11:** Table corresponding to Fig 13

	F3	predicted	std.error	conf.low	conf.high	Threat	F2
1	maintain	6.148	0.151	5.852	6.443	no/small	US supply
2	increase independently	6.236	0.155	5.931	6.540	no/small	US supply
3	increase cooperation	6.820	0.153	6.519	7.120	no/small	US supply
4	integrated army	5.928	0.155	5.625	6.231	no/small	US supply
5	maintain	5.320	0.161	5.004	5.636	no/small	US withdraw
6	increase independently	5.354	0.173	5.015	5.693	no/small	US withdraw
7	increase cooperation	6.313	0.168	5.984	6.642	no/small	US withdraw
8	integrated army	5.848	0.170	5.514	6.182	no/small	US withdraw
9	maintain	6.177	0.066	6.047	6.307	moderate	US supply
10	increase independently	6.492	0.067	6.362	6.623	moderate	US supply
11	increase cooperation	6.899	0.067	6.767	7.030	moderate	US supply
12	integrated army	6.417	0.066	6.288	6.546	moderate	US supply
13	maintain	5.301	0.074	5.155	5.446	moderate	US withdraw
14	increase independently	5.821	0.073	5.679	5.963	moderate	US withdraw
15	increase cooperation	6.336	0.073	6.194	6.478	moderate	US withdraw
16	integrated army	5.969	0.072	5.827	6.110	moderate	US withdraw
17	maintain	6.119	0.108	5.908	6.331	large	US supply
18	increase independently	6.567	0.105	6.361	6.773	large	US supply
19	increase cooperation	6.602	0.103	6.400	6.803	large	US supply
20	integrated army	6.398	0.107	6.188	6.608	large	US supply
21	maintain	5.228	0.110	5.011	5.444	large	US withdraw
22	increase independently	5.821	0.108	5.610	6.033	large	US withdraw
23	increase cooperation	6.108	0.107	5.898	6.318	large	US withdraw
24	integrated army	5.977	0.109	5.764	6.190	large	US withdraw

**Table 12:** Table corresponding to Fig 14 - ideology

	F3	predicted	std.error	conf.low	conf.high	model
1	maintain	5.924	0.089	5.750	6.098	left
2	increase independently	5.936	0.089	5.760	6.111	left
3	increase cooperation	6.941	0.087	6.770	7.112	left
4	integrated army	6.447	0.089	6.273	6.621	left
5	maintain	5.707	0.055	5.601	5.814	center
6	increase independently	6.052	0.054	5.946	6.158	center
7	increase cooperation	6.440	0.054	6.333	6.546	center
8	integrated army	6.155	0.054	6.050	6.261	center
9	maintain	5.688	0.086	5.519	5.857	right
10	increase independently	6.554	0.087	6.383	6.725	right
11	increase cooperation	6.424	0.085	6.258	6.591	right
12	integrated army	5.958	0.086	5.789	6.128	right

**Table 13:** Table corresponding to Fig 14 - identity

	F3	predicted	std.error	conf.low	conf.high	model
1	maintain	6.040	0.252	5.545	6.534	EU only
2	increase independently	6.292	0.250	5.803	6.781	EU only
3	increase cooperation	7.019	0.261	6.507	7.530	EU only
4	integrated army	6.297	0.240	5.827	6.767	EU only
5	maintain	6.121	0.094	5.938	6.305	EU & Nat.
6	increase independently	6.021	0.094	5.837	6.205	EU & Nat.
7	increase cooperation	7.180	0.092	7.000	7.359	EU & Nat.
8	integrated army	6.791	0.095	6.605	6.977	EU & Nat.
9	maintain	5.802	0.056	5.693	5.911	Nat. & EU
10	increase independently	6.358	0.055	6.249	6.466	Nat. & EU
11	increase cooperation	6.792	0.055	6.684	6.900	Nat. & EU
12	integrated army	6.420	0.055	6.313	6.527	Nat. & EU
13	maintain	5.366	0.090	5.190	5.543	Nat. only
14	increase independently	5.937	0.092	5.757	6.117	Nat. only
15	increase cooperation	5.684	0.089	5.509	5.859	Nat. only
16	integrated army	5.268	0.092	5.089	5.448	Nat. only

## F Data on Defense Spending

In Figs. 26 and 26, we present data on defense spending from the SIPRI Military Expenditure Database^12^. We show how military expenditures differ between smaller Central and Eastern European (CEE) countries and bigger Western European countries. CEE countries chose to invest more heavily in their national armies than their Western European counterparts. After 2014, CEE states bordering Russia choose to rather invest into national capacity. In Fig. 26 this is the most clearly visible if we restrict our sample to the Baltic countries and Poland. Note that at the beginning of the graph, defense spending is very low due to the fact that Baltic states were new states and were building their armies from scratch financially. Figure 27 confirms that even if we enlarge the sample to all CEE states, those would still rather build national capacity-see how average spending moved up after 2014 when Russian invaded Ukraine, while defense budgets for the West barely moved. In the main text, we argue that this is because CEE states find it more efficient to immediately invest into their own defense capacity and build their own defense industry than invest into pooled European military capacity which would be costly in terms of time and transaction costs (negotiations, implementation, raising European taxes...).

## Footnotes

1 When asked, a plurality, 32% of our respondents thought that the war in Ukraine was the most important threat to the survival of the EU, with a further 21% citing it as the second most important threat. Other option categories included climate change, financial crises, refugee inflows from outside Europe, refugee inflows from within Europe, member states leaving the EU, poverty and unemployment, and pandemics.

2 https://www.bbc.com/news/world-europe-60977917.

3 https://www.ft.com/content/9de49e7a-d830-4d5d-8615-2c00ba6f8552.

4 https://www.theguardian.com/us-news/2024/feb/15/trump-russia-attack-nato-campaign-biden.

5 Our argument regarding the long-term benefits of centralization rests on the increased efficiency resulting from a common “command and control” of a unified EU army. We chose a simple and intuitive wording of this scenario, as respondents should be aware of what a unified army implies in any type of state, be it unitary or federal. Furthermore, we chose nearly identical wording in the first part of the scenario with the “increased cooperation” scenario (see Table 1), so that respondents understand that both scenarios imply an increase of resources at the EU level. The difference between the two scenarios, therefore, rests on the idea of a integrated army.

6 One concern might be that respondents do not associate an “integrated army” with more centralized “command and control” and might therefore assume that individual member states can veto decisions, as in the status quo. However, the negative effect of the army scenario, compared to the coordination scenario, suggests this is not the case. An integrated army implies more “command and control”, or at the very least as much command and control as increased cooperation. We later present other empirical results that support this interpretation.

7 This finding supports our interpretation that respondents correctly understand that an EU army implies more “command and control” than increasing cooperation, and thus less sovereignty. To further test this possibility, we run an additional heterogeneous effect with a question asking individuals if they support the right of individual member states to veto EU decisions in foreign policy (see wording in Appendix B.2 Question wording). Figure 21 in the Appendix shows that the pro-veto group has a substantially lower rating of the “army” scenario than the anti-veto group, suggesting that indeed respondents understood that the army scenario implies a degree of command and control. We also see that the pro-veto group rated the “increase independently” and “cooperation” scenarios higher than the status quo, but not the ”army“ scenario.

8 https://www.ft.com/content/66606e2c-0b5a-493e-af59-d24436d0fd72.

9 https://www.bloomberg.com/news/articles/2024-02-26/eu-s-new-defense-plan-aims-to-boost-european-military-purchases.

10 https://www.bloomberg.com/news/articles/2023-03-24/nordic-nations-agree-to-jointly-operate-fighter-jet-fleet-of-250.

11 https://www.theguardian.com/world/2024/feb/12/european-leaders-call-for-stronger-defense-ties-donald-trump-nato-remarks-russia.
